# Genetic Variation and Population Structure of *Oryza glaberrima* and Development of a Mini-Core Collection Using DArTseq

**DOI:** 10.3389/fpls.2017.01748

**Published:** 2017-10-17

**Authors:** Marie-Noelle Ndjiondjop, Kassa Semagn, Arnaud C. Gouda, Sèdjro B. Kpeki, Daniel Dro Tia, Mounirou Sow, Alphonse Goungoulou, Moussa Sie, Xavier Perrier, Alain Ghesquiere, Marilyn L. Warburton

**Affiliations:** ^1^Africa Rice Center (AfricaRice), Bouake, Cote d'Ivoire; ^2^Department of Agriculture, Forestry and Nutrition Science, University of Alberta, Edmonton, Canada; ^3^Africa Rice Center (AfricaRice), Ibadan, Nigeria; ^4^Africa Rice Center (AfricaRice), Centre National de la Recherche Appliquée au Développement Rural (FOFIFA), Antananarivo, Madagascar; ^5^Unité Mixte de Recherche Amélioration Génétique, CIRAD, Montpellier, France; ^6^University of Montpellier, Montpellier, France; ^7^Plant Diversity Adaptation and Development Research Unit, Institut de Recherche pour le Développement - Université de Montpellier, Montpellier, France; ^8^Corn Host Plant Resistance Research Unit, United States Department of Agriculture, Agricultural Research Service, Starkville, Mississippi, United States

**Keywords:** African rice, plant genetic resources, *O. glaberrima*, core collection, genetic differentiation, genetic diversity, SNP

## Abstract

The sequence variation present in accessions conserved in genebanks can best be used in plant improvement when it is properly characterized and published. Using low cost and high density single nucleotide polymorphism (SNP) assays, the genetic diversity, population structure, and relatedness between pairs of accessions can be quickly assessed. This information is relevant for different purposes, including creating core and mini-core sets that represent the maximum possible genetic variation contained in the whole collection. Here, we studied the genetic variation and population structure of 2,179 *Oryza glaberrima* Steud. accessions conserved at the AfricaRice genebank using 27,560 DArTseq-based SNPs. Only 14% (3,834 of 27,560) of the SNPs were polymorphic across the 2,179 accessions, which is much lower than diversity reported in other *Oryza* species. Genetic distance between pairs of accessions varied from 0.005 to 0.306, with 1.5% of the pairs nearly identical, 8.0% of the pairs similar, 78.1% of the pairs moderately distant, and 12.4% of the pairs very distant. The number of redundant accessions that contribute little or no new genetic variation to the *O. glaberrima* collection was very low. Using the maximum length sub-tree method, we propose a subset of 1,330 and 350 accessions to represent a core and mini-core collection, respectively. The core and mini-core sets accounted for ~61 and 16%, respectively, of the whole collection, and captured 97–99% of the SNP polymorphism and nearly all allele and genotype frequencies observed in the whole *O. glaberrima* collection available at the AfricaRice genebank. Cluster, principal component and model-based population structure analyses all divided the 2,179 accessions into five groups, based roughly on country of origin but less so on ecology. The first, third and fourth groups consisted of accessions primarily from Liberia, Nigeria, and Mali, respectively; the second group consisted primarily of accessions from Togo and Nigeria; and the fifth and smallest group was a mixture of accessions from multiple countries. Analysis of molecular variance showed between 10.8 and 28.9% of the variation among groups with the remaining 71.1–89.2% attributable to differences within groups.

## Introduction

The ability of individuals, populations or species to adapt to changing environments is directly dependent on the amount of genetic variation (diversity) they possess (Frankham, 2005). In general, higher genetic diversity provides greater ability to adapt to changes in environmental conditions, while lower genetic diversity results in uniformity and reduces adaptability (Luan et al., 2006), which in turn increases the chance for extinction. Furthermore, plant breeders need sequence variation in breeding populations to accelerate gain from selection to improve yield, resistance to biotic and abiotic stresses, and other traits. Some of this genetic variation is present in genebank accessions, and may be used if properly cataloged; this may include finding specific new traits, or selection of parental combinations for developing progeny with maximum genetic variability for further selection (Barrett and Kidwell, 1998). Knowledge of genetic diversity and population structure is also useful for essential genebank tasks, including creating core and mini-core sets that represent the variation available in the whole collection, in a more manageable sample size (Yan et al., 2007; Agrama et al., 2009; El Bakkali et al., 2013); establishing benchmark data to serve as a baseline for estimating possible loss of genetic diversity during conservation (Reif et al., 2005); estimating the relative strengths of the evolutionary forces of genetic drift, natural selection, mutation, and gene flow on populations *ex situ* or *in situ* (Falconer and MacKay, 1996; Ouborg et al., 1999); and identifying gaps in genebank collections (Brown, 1989).

Genebanks aim to conserve germplasm that represent distinct combinations of genes and alleles that may confer adaptive potential for a wide range of economically important traits (McCouch et al., 2012). In most cases, however, only a small fraction of germplasm conserved at genebanks has been characterized in detail due to (i) technical and financial constraints in conducting detailed phenotypic evaluation of large germplasm collections in multiple environments; and (ii) lack of low cost, high-throughput, and high density molecular marker assays. In such cases, the development of core and mini-core sets would speed detailed phenotypic evaluation of target traits of interest under field or controlled conditions to provide information essential for breeding and gene discovery (Agrama et al., 2009; Tiwari et al., 2015). Different types of molecular markers have been used to characterize the genetic diversity and population structure of germplasm conserved in genebanks (Semagn et al., 2006; Sharma, 2017), but single nucleotide polymorphism (SNP) assays are popular due to their abundance, locus-specificity, low error rates, co-dominant inheritance, and potential for high throughput analysis (Rafalski, 2002; Schlotterer, 2004). SNP data can be obtained using numerous uniplex or multiplex genotyping platforms (Syvanen, 2001; Chen and Sullivan, 2003), but most are expensive for high density genotyping of large numbers of genebank accessions.

The recent development of next-generation sequencing based genotyping technologies, such as genotyping by sequencing (GBS) (Elshire et al., 2011) and the diversity arrays technology-based sequencing (DArTseq) platform (Sansaloni et al., 2011) generate high density SNP information with significantly reduced genotyping cost, allowing detailed molecular characterization of germplasm conserved in genebanks. While GBS has become a popular SNP genotyping method that generates up to a million SNPs per sample (Elshire et al., 2011; Crossa et al., 2013; Ertiro et al., 2015), the technology has some limitations, including a relatively large proportion of missing data points (Beissinger et al., 2013; Nazzicari et al., 2016), and high allele calling error-rates due to low read coverage and when genotyping heterogeneous and highly heterozygous germplasm (Semagn et al., 2015). Some of these drawbacks have been dealt with through intensive post data correction, including implementation of reliable imputation methods, re-analyzing/re-examining old data using improved computational tools, and increasing read coverage (Semagn et al., 2015; Furuta et al., 2017). DArTseq has been used as an alternative to GBS; while it generates lower density SNP information (ranging from a few thousand to 350,000 SNPs depending on the species), it has relatively better coverage and lower levels of missing data (Chen et al., 2016). The DArTseq assay has been used for studies of several species, including *Oryza sativa* spp. japonica (Courtois et al., 2013), *Zea mays* L (Chen et al., 2016; dos Santos et al., 2016), *Triticum turgidum* L. (Baloch et al., 2017), 16 taxonomic units of the genus *Secale* (Al-Beyroutiová et al., 2016), *Citrullus lanatus* (Thunb.) Matsum. and Nakai (Ren et al., 2015; Yang et al., 2016), and 13 genotypes from the genus *Ananas* that represent from primitive wild accessions to modern cultivars (Kilian et al., 2016), among others.

Rice is the second largest crop in total global production after maize (http://www.fao.org/faostat/en/#data; accessed in Sept. 2017) and feeds more people than any other crop (Maclean et al., 2002). It belongs to the genus *Oryza*, which consists of several wild and two cultivated species - the African rice (*O. glaberrima* Steud.) and the Asian rice (*O. sativa* L.). Although the International Rice Research Institute (IRRI) genebank in the Philippines conserves the largest collection of rice germplasm, the Africa Rice Center (AfricaRice) genebank also conserves approximately 22,000 registered rice samples that represent five African indigenous wild species (*O. barthii, O. longistaminata, O. eichingery, O. punctate*, and *O. branchyata*), as well as both modern and traditional *O. glaberrima* and *O. sativa* varieties. *O. glaberrima* accounts for approximately 14% of the collection at the AfricaRice genebank. Efforts have been made to characterize the *O. glaberrima* germplasm conserved at the AfricaRice genebank using phenotypic traits (Montcho et al., 2013; Mokuwa et al., 2014), molecular markers (Joshi et al., 2000; Ishii et al., 2001; Park et al., 2003; Semon et al., 2005; Kwon et al., 2006; Dramé et al., 2011; Mokuwa et al., 2014; Orjuela et al., 2014; Meyer et al., 2016), and DNA sequencing (Li et al., 2011; Wang et al., 2014). However, most of the previous genetic characterization studies were done on small numbers of accessions using few phenotypic traits and/or molecular markers. To improve characterization of the collection, the AfricaRice genebank used DArTseq to determine the genetic variation and population structure of the entire rice collection conserved in its genebank. This information is being used to create core and mini-core sets of germplasm representing the entire gene pool of each *Oryza* species conserved in the genebank; and to develop quality control genotyping methods to track seed identity, genetic purity, and minimize human error, including stray pollen contamination during regeneration, undesired mixing of seed lots, and labeling/handling errors. The objectives of the present study were to use the DArTseq data to (i) investigate the genetic variation, relatedness and population structure of *O. glaberrima* collection available at the AfricaRice genebank and (ii) create core and mini-core sets of accessions that capture most of the genetic variation of the whole *O. glaberrima* collection conserved at the AfricaRice genebank. This will aid breeders to efficiently tap the available sequence diversity of the collection to create improved cultivars.

## Materials and methods

### Plant materials and phenotyping

A total of 2,223 cultivated *O. glaberrima* accessions conserved at the AfricaRice (previously called the West Africa Rice Development Association, WARDA), genebank was initially used in this study (Supplementary Table S1). The accessions originated from 23 countries, with each country represented from one accession each from the Democratic Republic of Congo and Burundi to 575 accessions from Nigeria. Between 1973 and 1989, AfricaRice received germplasm collections for several *Oryza* species from the Office de la Recherche Scientifique et Technique d'Outre-Mer (ORSTOM) in France, Institute de Recherche d'Afrique Tropicale (IRAT) in France, the International Institute for Tropical Agriculture (IITA) in Nigeria, the Central Agricultural Research Institute (CARI) in Liberia, and the Kogoshima University in Liberia. Additional collections were made by AfricaRice scientists and collaborators to fill collection gaps of the different *Oryza* species growing across floating, mangrove, swamp, hydromorphic, irrigated lowland, rainfed lowland, and upland growing ecologies in Africa. Nearly 92% of the 2,223 accessions used in the present study originate from eight countries, which includes Nigeria (25.9%), Liberia (23.5%), Mali (12.2%), Togo (10.3%), Guinea Conakry (6.8), Côte d'Ivoire (Ivory Coast) (4.9%), Senegal (4.8%) and Burkina Faso (3.3%) (Supplementary Table S1). AfricaRice was created in the early 1970s in Monrovia, Liberia. Because of the civil disturbances in Liberia, however, AfricaRice moved its headquarter from Liberia to the Ivory Coast in 1987/1988. During the civil disturbance and relocation period, part of the passport data was lost, which forced the AfricaRice genebank staff to register several accessions with missing passport data either to Liberia (the former AfricaRice head office) or Nigeria where many rice collections were received from IITA, which may have contributed to the larger proportion of accessions originated from both Liberia and Nigeria as compared with all other countries.

To update the phenotypic descriptors of the whole *O. glaberrima* collection by growing them in the same environmental conditions, we evaluated each accession in a three-row plot of 0.4 m wide × 1.0 m long under sprinkler irrigation at the AfricaRice experimental field in Cotonou, Benin (6° 25′ N latitude and at 2° 20′ E longitude). Seeds were directly sown in the field with a spacing of 0.20 m between rows and 1 m between accessions in August 2016 using randomized incomplete block design with a single replication; standard agronomic practices and inputs were used. For each accession, seven phenotypic traits were recorded on 5 randomly selected plants from the inner row of each plot using the Standard Evaluation System for Rice (http://www.knowledgebank.irri.org/ses/SES.htm; accessed in Sept. 2017). Days to heading was recorded as the number of days from seeding until 50% of the panicles had headed. Total number of tillers per plant was counted at maximum tillering, while number of fertile tillers per plant were counted at maturity when about 50% of the panicles in a plot lost their greenness. Plant height was measured from the base of the plants to the tip of heads, excluding awns, at maturity. Panicle length was measured from the panicle neck to the panicle tip, excluding awns, at maturity.

### DNA extraction and genotyping

Genomic DNA was extracted from a single plant per accession from screenhouse grown 3-weeks old seedling in 2016 using the cetyltrimethyl ammonium bromide (CTAB) method (Murray and Thompson, 1980), with minor modifications. DNA concentration was measured using a SmartSpec Plus spectrophotometer (Bio-Rad, USA) as described in the user's manual, and normalized to 100 ng/μL. DNA samples were shipped to DArT Pty Ltd, Canberra, Australia and genotyped in 96-plex using the DArTseq^TM^ technology (Sansaloni et al., 2011; Ren et al., 2015). The detailed methodology on complexity reduction, cloning, library construction and cleaning are described in a recent paper (Egea et al., 2017). Amplification fragments were sequenced on the Illumina Hiseq 2500 (www.illumina.com) and SNPs were called using the DArTsoft analytical pipeline (http://www.diversityarrays.com/dart-technology-package-dartSoft). A total of 31,739 SNPs were successfully called, of which approximately 18% of the SNPs had missing data points ranging from 0 to 79.7%, with an overall average missing data points of 11% (data not shown). The missing alleles of SNPs with <50% missing data points (Supplementary Table S1, Supplementary Figure S1a) were imputed by DArT Pty Ltd as described in another study (Swarts et al., 2014). A total of 27,560 imputed SNPs (Table 1) were then received from DArT Pty Ltd that were polymorphic across 5 *Oryza* species sampled from the AfricaRice genebank, each SNP with a minor allele frequency of > 0.01.

**Table 1:** The chromosomal distribution of 3,834 polymorphic SNPs used for genotyping 2,179 *O. glaberrima* accessions, including the physical length of each chromosome covered by the SNPs (in kilobase pairs) and the average distance between SNPs.

**Chromosomes**	**Physical length based on 27,560 SNPs (kb)**	**Initial number of polymorphic SNPs used for genotyping**	**Number of SNPs polymorphic in 2,179 *O. glaberrima***	**Average map length per SNP (kb)**
Chr1	43,230	2,731	308	140
Chr2	35,885	2,446	285	126
Chr3	36,413	2,318	257	142
Chr4	35,498	2,062	239	149
Chr5	29,763	1,681	214	139
Chr6	31,191	1,866	235	133
Chr7	29,679	1,780	228	130
Chr8	28,429	1,654	149	191
Chr9	22,947	1,375	158	145
Chr10	23,205	1,444	224	104
Chr11	29,000	1,839	317	91
Chr12	27,505	1,719	233	118
Unmapped	–	4,645	987	–
Total	372,746	27,560	3,834	

### Statistical analyses

For each SNP, polymorphism information content (PIC) values were computed using PowerMarker v.3.25 (Liu and Muse, 2005). The proportion of observed heterozygosity in each accession was calculated using TASSEL v.5.2.37 (Bradbury et al., 2007). A genetic distance matrix was calculated between each pair of accessions using the identity-by-state (IBS) method implemented in TASSEL v.5.2.37. Cluster analysis was performed on the genetic distance matrix using the neighbor-joining algorithm implemented in molecular evolutionary genetics analysis (MEGA) v.6.0 (Tamura et al., 2013) and the Dissimilarity Analysis and Representation for windows (DARwin) v.6.0.14 (Perrier et al., 2003). Population structure was evaluated using the model-based method implemented in the software package STRUCTURE v.2.3.4 (Pritchard et al., 2000). STRUCTURE was run by varying the number of clusters (K) from 1 to 10 as described elsewhere (Semagn et al., 2012b, 2014); each K was repeated three times with a burn-in period of 10,000 and 10,000 MCMC (Markov Chain Monte Carlo) replications after burn-in. For each *K*-value, genotypes with probability of membership >60% were assigned to the same group, while those with <60% probability in any group were assigned to a “mixed” group (Yang et al., 2011; Semagn et al., 2012b). Principal component analysis (PCA) was performed using TASSEL v.5.2.37. The first two principal components from the PCA were plotted for visual examination in XLSTAT 2012 (Addinsof, New York, USA; www.xlstat.com) using the scatter plot option with the following categorical variables: countries of origin and ecologies of accessions, and group memberships obtained from cluster analysis and the model-based STRUCTURE.

The extent of polymorphism, heterozygosity, nucleotide diversity, and F_ST_-based pairwise genetic distance matrices (Holsinger and Weir, 2009) were computed among categorical variables using ARLEQUIN v.3.5.2.2 (Excoffier and Lischer, 2010). *F*_*ST*_ values are indicative of the evolutionary processes that influence the structure of genetic variation among populations or groups, with <0.05 indicating little, 0.05–0.15 moderate, 0.15–0.25 great, and > 0.25 very great genetic differentiation (Wright, 1978). Analysis of molecular variance (AMOVA) (Excoffier et al., 1992) was also computed using ARLEQUIN v.3.5.2.2 to partition the variation among and within groups of accessions belonging to the different categorical variables listed above.

The maximum (core) and minimum (mini-core) number of *O. glaberrima* accessions required to capture the majority of alleles and genetic diversity observed in the whole *O. glaberrima* collection were found using the maximum length sub-tree (MLST) method implemented in DARwin v.6.0.14. Sphericity index (the ratio of the lengths of external edges to the total length of the sub tree) was used in deciding the most informative number of genotypes to be retained or removed. In the presence of redundant genotypes, the sphericity index curve would be linear (in a plateau) with the initial value obtained before any genotype is removed, which is indicative of the number of redundant genotypes to be removed. The curve increases as each redundancy is removed until it reaches another plateau, at which point the minimum number of genotypes that can be retained while conserving maximum diversity has been reached (Perrier et al., 2003). The phenotypic traits were summarized using MiniTab v14.

## Results

### Initial analyses and marker polymorphism

**Figure 1:**
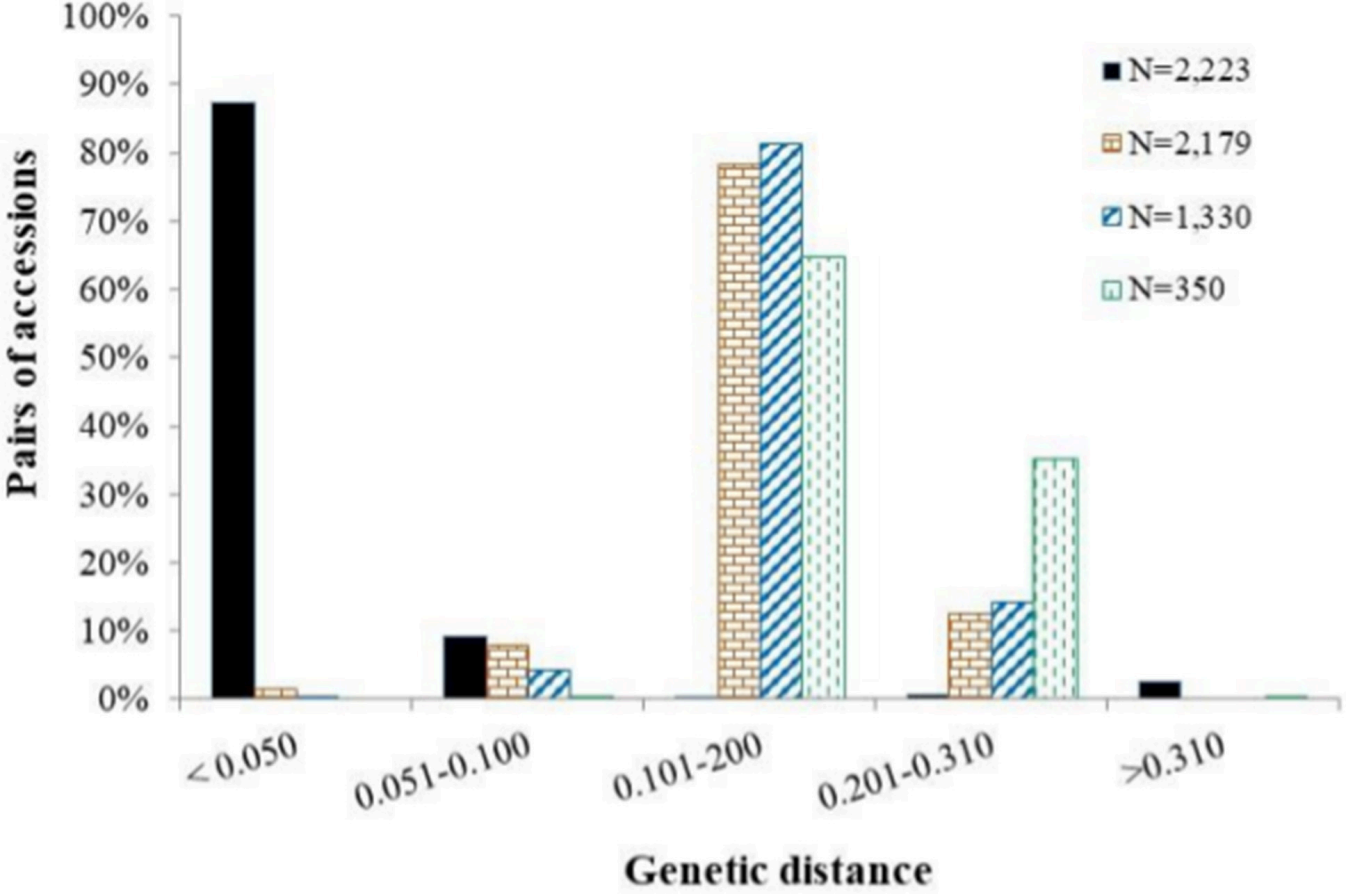
Frequency distribution categories of pairwise genetic distance of 2,223 *O. glaberrima* accessions based on 16, 532 SNPs (black fill); 2,179 accessions (after excluding the 44 most diverse accessions) based on 3,834 SNPs (orange brick fill); a core set of 1,330 accessions (blue diagonal line fill) based on 3,781 polymorphic SNPs, and a mini-core set of 350 accessions (green dashed line fill), based on 3,709 polymorphic SNPs.

Of the 27,560 SNPs (Table 1) that were polymorphic across 5 *Oryza* species, nearly 60% (16,532 SNPs) were polymorphic across the 2,223 *O. glaberrima* accessions. Minor allele frequency in the *O. glaberrima* data set ranged from 0.010 to 0.498 (Supplementary Figure S1c). Approximately 46% of the polymorphisms (12,698 out of the 27,560 SNPs) were found in only 44 accessions originated from 11 countries (see list of accessions at the end of Supplementary Table S1) that showed extremely different haplotypes as compared with all other *O. glaberrima* accessions. Genetic distance between pairs of the 2,223 accessions computed from the 16,532 polymorphic SNPs varied from 0.002 to 0.841, with an overall average of 0.058. The majority (87.4%) of the pairs of accessions had a genetic distance of < 0.05, which is extremely small as compared with just 2.7% of the pairs of accessions that had genetic distance > 0.300 (Figure 1). A neighbor-joining tree generated from the genetic distance matrix of the 16,532 SNPs revealed radical separation of the 44 accessions from all others (Supplementary Figure S2). Because of this distance, and the possibility that these accessions may be natural introgressions from other *Oryza* species, or have been misclassified, they were excluded from further analysis. Following exclusion, only 3,834 SNPs (13.9%) were polymorphic among the remaining 2,179 accessions (Table 2), each with minor allele frequency ranging from 0.010 to 0.498 (Supplementary Figure S1c). Polymorphism information content of each of the 3,834 SNP was fairly low, varying from 0.020 to 0.375 (Supplementary Figure S3). Approximately 74% of the 3,834 polymorphic SNPs were mapped onto one of the 12 rice chromosomes, and the remaining 26% were unmapped (Table 1). The number of mapped SNPs varied from 149 on chromosome 8 to 317 on chromosome 11, with an overall average of 237 SNPs per chromosome. The physical length of each chromosome varied from 22,947 kb on chromosome 9 to 43,230 kb on chromosome 1 (Table 1, Supplementary Figure S4) and the overall genome size was 372.7 Mb.

**Table 2:** Summary of the 2,179 *O. glaberrima* accessions used in the present study and the number of accessions selected to represent a core and mini-core sets.

**Country of origin**	**Final number of accessions used**	**No. of accessions retained in the core set**	**No. of accessions retained in the mini-core set**	**No. of accessions belonging to each group based on STRUCTURE at *K* = 5**						**No. of accessions belonging to each group based on cluster analysis**				
				**Group 1**	**Group 2**	**Group 3**	**Group 4**	**Group 5**	**Admixture**	**Group 1**	**Group 2**	**Group 3**	**Group 4**	**Group 5**
Benin	3	2	2	–	–	1	1	–	1	–	1	1	1	–
Burkina Faso	74	59	14	3	3	–	44	20	4	2	7	2	51	12
Burundi	1	1	1	–	–	–	–	–	1	–	–	1	–	–
Cameroon	37	26	7	1	30	3	1	2	–	1	33	1	–	2
Chad	18	13	2	–	13	–	2	3	–	–	15	–	1	2
Congo	1	–	–	–	–	–	–	1	–	–	–	–	–	1
Egypt	3	1	1	–	1	1	–	1	–	–	2	–	–	1
Gambia	24	18	7	1	9	7	1	4	2	–	16	1	2	5
Ghana	37	29	2	1	23	1	4	7	1	1	26	1	7	2
Guinea Conakry	145	92	19	8	5	2	22	92	16	11	12	4	49	69
Guinea Bissau	14	12	3	–	–	1	2	11	–	–	1	1	1	11
Ivory Coast	104	67	15	10	13	5	27	45	4	10	40	8	29	17
Liberia	518	188	33	300	19	11	19	157	12	315	54	14	25	110
Madagascar	3	3	–	–	1	–	2	–	–	–	1	–	2	–
Mali	267	218	76	14	14	6	211	15	7	13	18	6	218	12
Niger	6	6	3	–	–	2	3	–	1	–	–	2	4	–
Nigeria	563	410	136	30	191	232	16	47	47	31	230	251	23	28
Senegal	99	76	20	10	1	5	7	67	9	10	12	6	11	60
Sierra Leone	7	3	1	2	–	1	–	4	–	3	–	1	–	3
Tanzania	4	4	1	–	1	1	1	1	–	–	1	1	1	1
Togo	226	86	2	4	210	1	2	9	–	4	214	–	2	6
Zambia	4	4	1	–	1	–	3	–	–	–	1	–	3	–
Zimbabwe	2	1	–	1	–	–	–	1	–	1	–	–	–	1
Others	19	11	4	2	1	3	6	6	1	2	2	2	8	5
Total	2,179	1,330	350	387	536	283	374	493	106	404	686	303	438	348

### Genetic purity, distance, and relationships

Observed heterozygosity per accession ranged from 0 to 19.3% (Supplementary Figure S5), with an overall average of 0.8%. Only 114 accessions (5.2% of the 2,179 accessions) had observed heterozygosity > 3.1% (Supplementary Table S1). Genetic distance between pair of the 2,179 accessions ranged from 0.005 to 0.306 (Supplementary Table S2), and the overall average distance was 0.153. As shown in Figure 1 and Supplementary Table S2, the majority (78.1%) of the pairs of accessions had a genetic distance value that fell between 0.10 and 0.20 as compared to the 9.5 and 12.5% that fellow below 0.10 and greater than 0.20, respectively.

**Figure 2:**
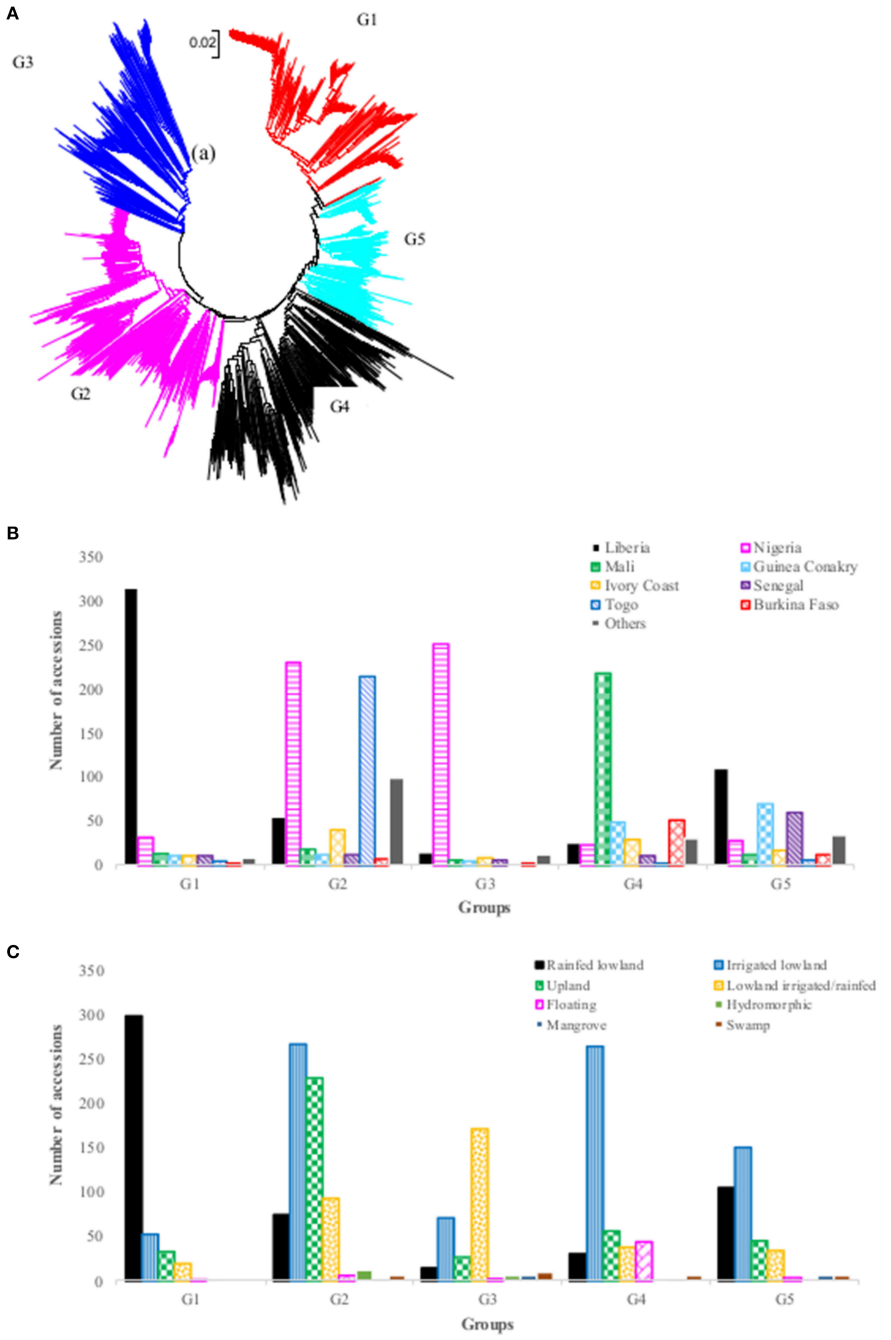
**(A)** Neighbor-joining tree of 2,179 *Oryza glaberrima* accessions based on 3,834 polymorphic SNPs (G1 = Group 1, red font; G2 = Group 2, pink font; G3= Group 3, blue font; G4 = Group 4, black font; G5 = Group 5, aqua font); **(B)** summary of accessions in each group by country of origin (only countries with >40 accessions were listed here); **(C)** summary of accessions belonging to each group by ecology. See Supplementary Table S1 for detail group membership.

The neighbor-joining cluster analysis performed on the genetic distance matrix grouped the 2,179 accessions into five groups (Figure 2A, Supplementary Table S1). The groups clustered somewhat according to geography and country of origin. The first group consisted of 404 accessions originating from 12 countries, but the vast majority (77.9%) was from Liberia. The second group consisted of 686 accessions from 18 countries, but most accessions (65%) were from Nigeria (33.5%) and Togo (31.2%). The third group consisted of 303 accessions originating from 16 countries, of which 82.8% were from Nigeria. Group four had a total of 438 accessions from 17 countries, but Mali accounted for half (49.8%), followed by smaller contributions of over 5% from five other countries: Burkina Faso (11.6%), Guinea Conakry (11.2%), Ivory Coast (6.6%), Liberia (5.7%), and Nigeria (5.3%). The fifth group had 348 accessions from 18 countries and there was no clear majority from any single country; however, 92% of the accessions originated from 8 of 18 countries: Liberia (31.6%), Guinea Conakry (19.8%), Senegal (17.2%), Nigeria (8.0%), Ivory Coast (4.9%), Mali (3.4%), Burkina Faso (3.4%), and Guinea Bissau (3.2%) (Figure 2B, Supplementary Table S1). The clustering of accessions to the five groups also showed a pattern by ecology, although less clearly so (Figure 2C). The first group consisted of approximately 74% of the accessions from the rainfed lowland ecology; the second group was primarily a mixture of irrigated lowland and upland; the third group mostly rainfed and irrigated lowland; the fourth group mostly irrigated lowland, and the fifth group again a more balanced mixture of all ecologies (but more irrigated lowland than others).

### Population structure

**Figure 3:**
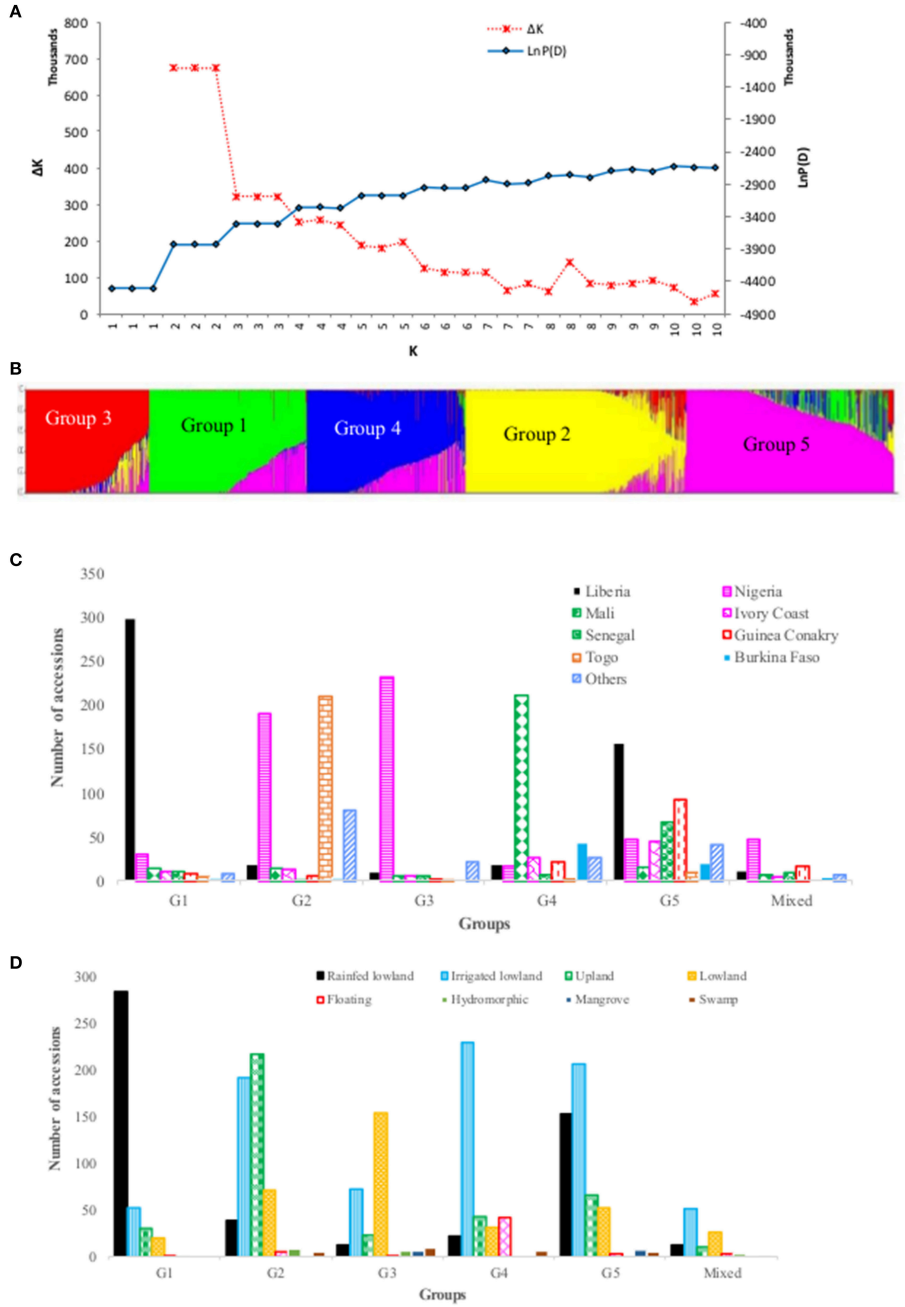
Population structure of 2,179 accessions based on 3,834 polymorphic SNPs: **(A)** plot of LnP(D) and an ad hoc statistic ΔK calculated for K ranging from 1 to 10, with each K repeated thrice; **(B)** population structure at *K* = 5, with each accession represented by a single vertical line that is partitioned into K colored segments, with lengths proportional to the estimated probability membership value (y-axis); **(C)** frequency distribution of accessions belonging to each predicted group by country of origin, with all countries that had <40 accessions combined in to “others”; **(D)** frequency distribution of accessions belong to each predicted group by ecology, with “others' referring to accessions from floating, mangrove, deep and shallow forest swamp, and hydromorphic ecologies. See Supplementary Table S1 for details.

**Figure 4:**
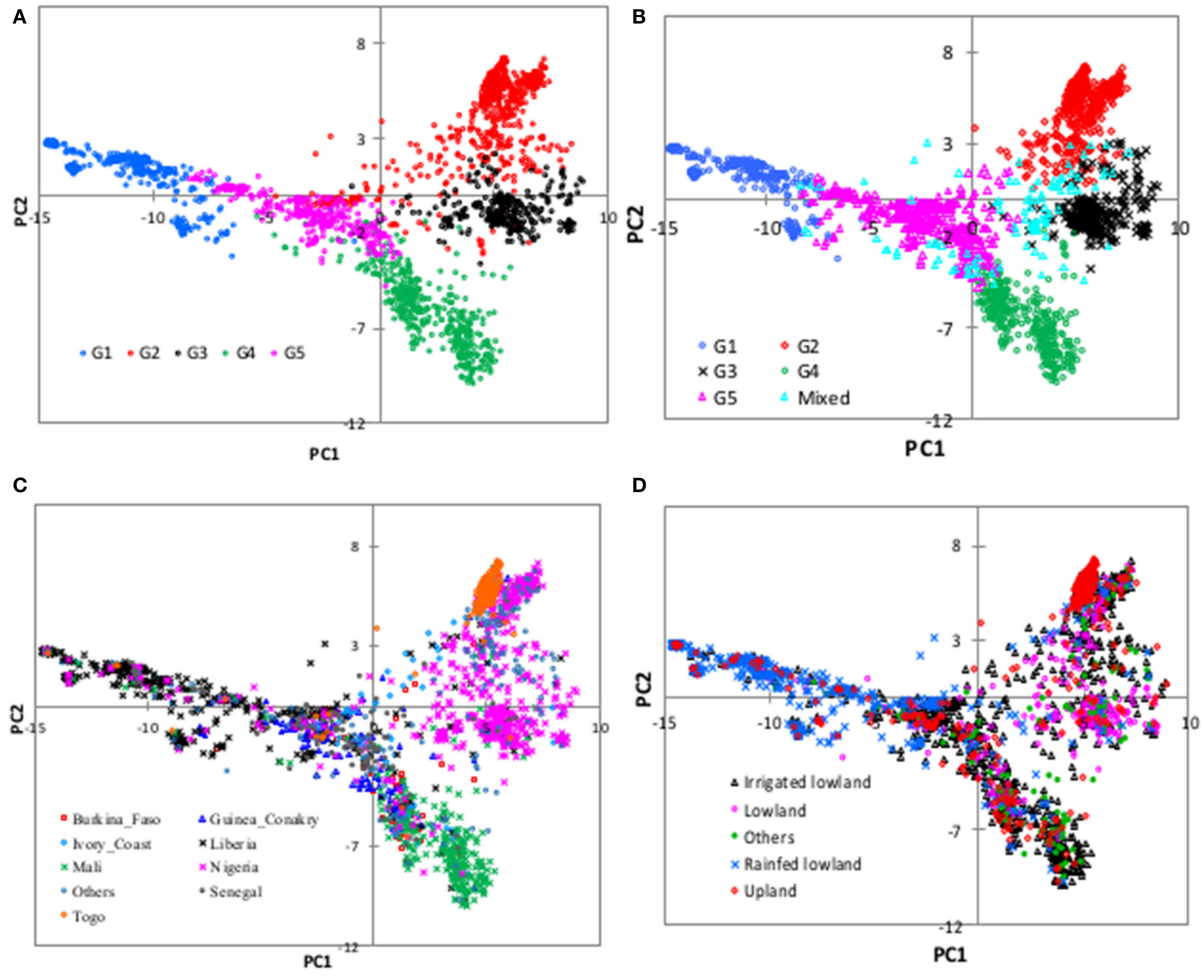
Plots of PC1 (14.6% of variation) and PC2 (5.8%) from principal component analyses of 2,179 *O. glaberrima* accessions genotyped with 3,834 polymorphic SNPs. The plots are based on group membership from **(A)** cluster analysis, **(B)** STRUCTURE at *K* = 5, **(C)** country of origin, and **(D)** ecology. All countries with < 40 accessions were combined to “others.” In the ecology plot, “others” refers to accessions from floating, mangrove, deep and shallow forest swamp, and hydromorphic ecologies, each with smaller number of accessions. See Supplementary Table S1 for details.

We explored population structure in the *O. glaberrima* germplasm conserved at the AfricaRice genebank using the model-based population structure. The log probability of the data (LnP(D)) increased between *K* = 1 and *K* = 5, and mostly reached a plateau between *K* = 5 and *K* = 10, while the ad hoc statistic ΔK declined between *K* = 2 and *K* = 5 (Figure 3), which suggest the presence of up to five possible groups in the germplasm. To decide the most likely number of groups that exist in the *O. glaberrima* collection, we used the group memberships from STRUCTURE in plotting the first two principal components from PCA analysis. A plot of PC1 (14.6% of the variation) and PC2 (5.8%) revealed 5 groups (Figure 4) with the same pattern of groupings as the model-based population partition at *K* = 5. Nearly all individuals assigned to a group at *K* = 5 belong to the same group in the principal component analysis, with the mixed group being intermediate between the second and third groups (Figure 4B). The proportion of accessions assigned to the five groups predicted from STRUCTURE varied from 13.0% in group 3 to 24.6% in group 2 (Supplementary Table S1). Group membership obtained from STRUCTURE agreed with that of cluster analysis for 1,868 accessions (85.7%); the remaining 311 accessions (14.3%) differed in their group membership between cluster analysis and STRUCTURE. Of these, 106 accessions were assigned in to a mixed group in STRUCTURE. The other 205 accessions grouped into different STRUCTURE groups than would have been predicted by cluster analysis, including accessions that belong to the STRUCTURE G1 (8 accessions), G2 (3 accessions), G3 (32 accessions), G4 (7 accessions) and G5 (155 accessions) (Supplemental Table S1). Excluding accessions that were assigned in to a mixed group, nearly 76% of the accessions that showed discrepancy between the cluster analysis and STRUCTURE came from group 5. The fifth group consisted of 493 accessions from 18 countries of which 89.9% were from seven countries—Liberia (31.8%), Guinea Conakry (18.7%), Senegal (13.6%), Nigeria (9.5%), Ivory Coast (4.1%), and Burkina Faso (3.0%) (Figure 3, Supplementary Table S1).

### Molecular diversity and genetic differentiation

Table 3 shows a summary of the percent polymorphism, observed heterozygosity, gene diversity, and nucleotide diversity among accessions belonging to the same categorical variables (country, ecology and predicted group membership based on cluster analysis and the model-based STRUCTURE). Of the 3,834 SNPs that were polymorphic across all 2,179 *O. glaberrima* accessions, the lowest (44.3%) and highest (97.8%) polymorphism was observed within accessions originating from Chad and Nigeria, respectively, which may be due mainly to the size of samples from each country. Nucleotide diversity varied from 0.055 for accessions originated from Togo to 0.161 for those from Mali. When polymorphism and nucleotide diversity among ecology groups were compared, polymorphism was the lowest in mangrove (52.8%) and highest in irrigated lowland (100%), while nucleotide diversity was the lowest in upland (0.118) and the highest (0.160) in lowland irrigated/rainfed ecologies. Accessions that belong to the first group predicted based on cluster analysis and the model-based STRUCTURE analysis showed the least polymorphism (71.2–73.9%), while those accessions in the third group had the highest gene and nucleotide diversity (Table 3).

**Table 3:** Summary of polymorphism and molecular diversity among accessions belonging to different hierarchical levels (country and ecology of origin of accessions plus predicted groups based on the model-based STRUCTURE at *K* = 5 and cluster analysis).

**Category**	**Description**	**No. of accessions**	**Polymorphism (%)**	**Average observed heterozygosity (Std.)**	**Gene diversity (Std.)**	**Average nucleotide diversity (Std.)**
**COUNTRY OF ORIGIN**						
	Burkina Faso	74	79.6	0.012 (0.038)	0.165 (0.156)	0.131 (0.062)
	Cameroon	37	67.0	0.021 (0.049)	0.175 (0.155)	0.117 (0.056)
	Chad	18	44.3	0.017 (0.057)	0.269 (0.151)	0.119 (0.058)
	Gambia	24	59.4	0.019 (0.049)	0.237 (0.164)	0.141 (0.068)
	Ghana	37	68.3	0.014 (0.040)	0.161 (0.132)	0.110 (0.053)
	Guinea Conakry	145	89.5	0.008 (0.030)	0.135 (0.138)	0.121 (0.057)
	Guinea Bissau	14	52.0	0.008 (0.042)	0.248 (0.135)	0.129 (0.063)
	Ivory Coast	104	93.0	0.008 (0.031)	0.150 (0.125)	0.139 (0.066)
	Liberia	518	97.7	0.010 (0.034)	0.117 (0.148)	0.114 (0.054)
	Mali	267	96.1	0.007 (0.031)	0.167 (0.154)	0.161 (0.076)
	Nigeria	563	97.8	0.008 (0.030)	0.161 (0.143)	0.157 (0.074)
	Senegal	99	86.4	0.013 (0.033)	0.136 (0.133)	0.117 (0.055)
	Togo	226	73.4	0.006 (0.034)	0.075 (0.126)	0.055 (0.026)
	Others	53	90.1	0.007 (0.030)	0.180 (0.125)	0.160 (0.077)
**ECOLOGY OF ORIGIN**						
	Floating	55	82.4	0.009 (0.035)	0.187 (0.151)	0.154 (0.073)
	Hydromorphic	18	55.4	0.027 (0.054)	0.251 (0.146)	0.139 (0.067)
	Irrigated lowland	804	100.0	0.008 (0.028)	0.154 (0.120)	0.154 (0.073)
	Lowland irrigated/rainfed	353	97.9	0.010 (0.029)	0.164 (0.139)	0.160 (0.076)
	Mangrove	13	52.8	0.011 (0.044)	0.285 (0.133)	0.151 (0.074)
	Rainfed lowland	521	97.6	0.010 (0.033)	0.121 (0.146)	0.119 (0.056)
	Swamp	26	66.8	0.020 (0.043)	0.232 (0.131)	0.155 (0.074)
	Upland	389	99.6	0.006 (0.028)	0.118 (0.119)	0.118 (0.056)
**GROUPS BASED ON CLUSTER ANALYSIS**						
	Group 1	404	71.2	0.013 (0.045)	0.129 (0.168)	0.092 (0.043)
	Group 2	686	93.8	0.009 (0.032)	0.114 (0.139)	0.107 (0.051)
	Group 3	303	84.3	0.012 (0.034)	0.189 (0.175)	0.159 (0.075)
	Group 4	438	92.6	0.008 (0.033)	0.167 (0.156)	0.155 (0.073)
	Group 5	348	72.2	0.007 (0.032)	0.122 (0.146)	0.088 (0.042)
**GROUPS BASED ON STRUCTURE AT *K* = 5**						
	Group 1	387	71.7	0.013 (0.046)	0.124 (0.166)	0.089 (0.042)
	Group 2	536	84.8	0.008 (0.034)	0.111 (0.151)	0.094 (0.044)
	Group 3	283	81.3	0.013 (0.036)	0.198 (0.178)	0.160 (0.076)
	Group 4	374	83.8	0.008 (0.034)	0.184 (0.161)	0.154 (0.073)
	Group 5	493	89.0	0.007 (0.029)	0.167 (0.136)	0.095 (0.045)
	Mixed	106	92.3	0.019 (0.034)	0.159 (0.126)	0.147 (0.069)

*All analyses were based on 2,179 O. glaberrima accessions genotyped with 3,834 polymorphic SNPs*.

When pairwise *F*_ST_ values were compared to understand the extent of genetic differentiation (divergence) between pairs of countries (Supplementary Table S3), the values were highly variable ranging from 0.020 (little genetic differentiation) to 0.420 (very great genetic differentiation). Accessions from Togo showed very great genetic differentiation (0.261–0.420) as compared with all other countries, except moderate differentiation from Ghana (0.115) and great genetic differentiation from Nigeria (0.188). *O. glaberrima* accessions from Liberia showed very great genetic differentiation from those from Cameroon (0.302), Chad (0.310), and Gambia (0.282), and great differentiation from those from Burkina Faso (0.221), Ghana (0.246) and Guinea Bissau (0.218). Accessions from Mali showed great genetic differentiation from those from Liberia (0.241), Cameroon (0.175) Chad (0.165) and Gambia (0.154), and moderate differentiation from all other countries. Similarly, accessions from Nigeria showed great differentiation from those accessions from Liberia (0.217) and moderate differentiation as compared with all other countries (Supplementary Table S3). Neighbor-joining cluster analysis performed on the pairwise *F*_ST_ values computed among pairs of countries showed three clusters, which corresponds to (i) the Liberia, Senegal, Guinea Conakry, Guinea Bissau, Burkina Faso and Mali group (although Liberia was a bit of an outlier); (ii) the Nigeria, Gambia, Cameroon and Chad group, and (iii) the Ghana and Togo group, which showed the greatest within-group differentiation (Supplementary Figure S6). The estimated pairwise fixation indices (*F*_*ST*_) among eight ecologies varied from 0.026 to 0.250 (Supplementary Table S3), of which the rainfed lowland ecology showed the highest genetic differentiation as compared to the floating (0.246), swamp (0.224), and hydromorphic (0.250) ecologies. Neighbor-joining cluster analysis performed on the pairwise *F*_ST_ values computed among pairs of ecologies revealed distinct clusters for the rainfed and upland ecologies as compared to all other ecologies (Supplementary Figure S6).

Results from partitioning of the overall molecular variance into different hierarchical levels revealed that differences in ecology and country of origin accounted for 10.8 and 18.1% of the genetic variation, respectively (Table 4). Higher among-groups variation was also observed when the variance components were partitioned based on the number of groups predicted using cluster analysis (27.3%) and the model-based STRUCTURE at *K* = 5 (28.9%) than either countries of origin or ecologies. Pairwise *F*_ST_ values among accessions that belong to group 1 predicted based on cluster analysis and the model-based STRUCTURE was the highest (0.268–0.469) as compared to the other four groups. A random permutation test indicated that the proportion of variance attributable for all hierarchical levels were highly significant (*p* < 0.001).

**Table 4:** Analysis of molecular variance (AMOVA) for the extraction of SNP variation among and within groups (populations) based on 2,179 *O. glaberrima* accessions genotyped with 3,834 polymorphic SNPs.

**Category**	**Source of variation**	**d.f**.	**Sum of squares**	**Variance components**	**Percentage of variation**
Country of origin^*^	Among groups	13	202,566.7	54.5	18.1
	Within groups	4,344	1,072,188.8	246.8	81.9
	Total	4,357	1,274,755.5	301.3	100.0
Ecology^*^	Among groups	7	107,343.2	32.4	10.8
	Within groups	4,350	1,167,789.9	268.5	89.2
	Total	4,357	1,275,133.1	300.9	100.0
Groups based on STRUCTURE at K = 5	Among groups	5	316,630.5	89.6	28.9
	Within groups	4,352	958,124.9	220.2	71.1
	Total	4,357	1,274,755.4	309.8	100.0
Groups based on cluster analysis	Among groups	4	289,596.6	84.8	27.3
	Within groups	4,353	985,158.8	226.3	72.7
	Total	4,357	1,274,755.4	311.1	100.0

* *Countries of origin: Burkina Faso, Cameroon, Chad, Gambia, Ghana, Guinea Conakry, Guinea Bissau, Ivory Coast, Liberia, Mali, Nigeria, Senegal, Togo, and “others,” which consisted of all other countries with <10 accessions. Ecologies of origin: Floating, Hydromorphic, Irrigated lowland, Lowland irrigated/rainfed, Rainfed lowland, Mangrove, Swamp, and upland*.

### Creating core and mini-core collections

**Figure 5:**
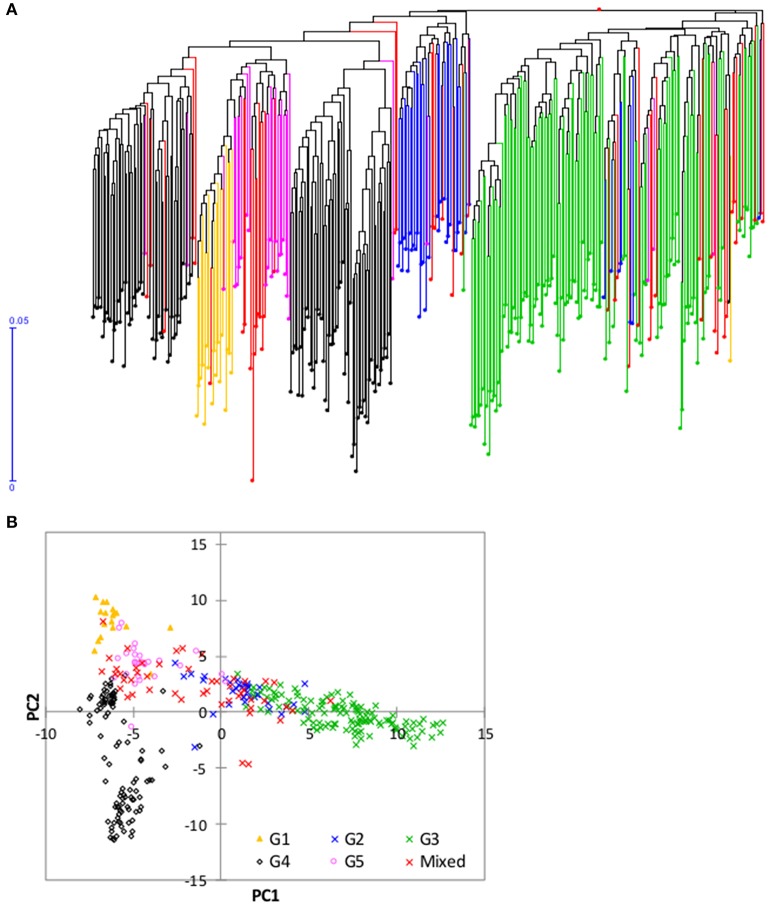
Neighbor joining tree and principal component analysis of the 350 mini-core accessions based on 3,709 of the 3,834 SNPs that were polymorphic within this set; **(A)** Neighbor joining tree from DARwin; and **(B)** plot of PC1 (10.6%) and PC2 (5.7%) from principal component analyses. Accessions belonging to each of the five groups predicted based on STRUCTURE in Figure 3 are shown with the same color (Group 1, orange font; Group 2, blue font; Group 3, green font; Group 4, black font; Group 5, pink font, and mixed, red font). See Supplementary Table S1 for details.

As shown in Supplementary Figure S7, the sphericity index was almost linear with relatively low slope until it reached to a sample size of 849 accessions on the x-axis, which suggests that the 849 accessions are not informative (highly similar with other accessions) and can be removed from the dataset without notably modifying the sphericity index (Supplementary Figure S8a). The remaining 1,330 accessions can be chosen to represent a core collection of *O. glaberrima* germplasm (Supplementary Figure S8b, Supplementary Table S1). The sphericity index increases rapidly between 849 to 1850 on the x-axis, which suggests that edges that constitute the backbone of the phylogenetic tree have been removed. Thus, the remaining 329 accessions could form the mini-core set. We propose retaining 350 accessions to represent a mini-core collection of the *O. glaberrima* germplasm (Figure 5, Supplementary Table S1). The selected core and mini-core sets originated from 22 and 20 countries, respectively (Table 2).

**Figure 6:**
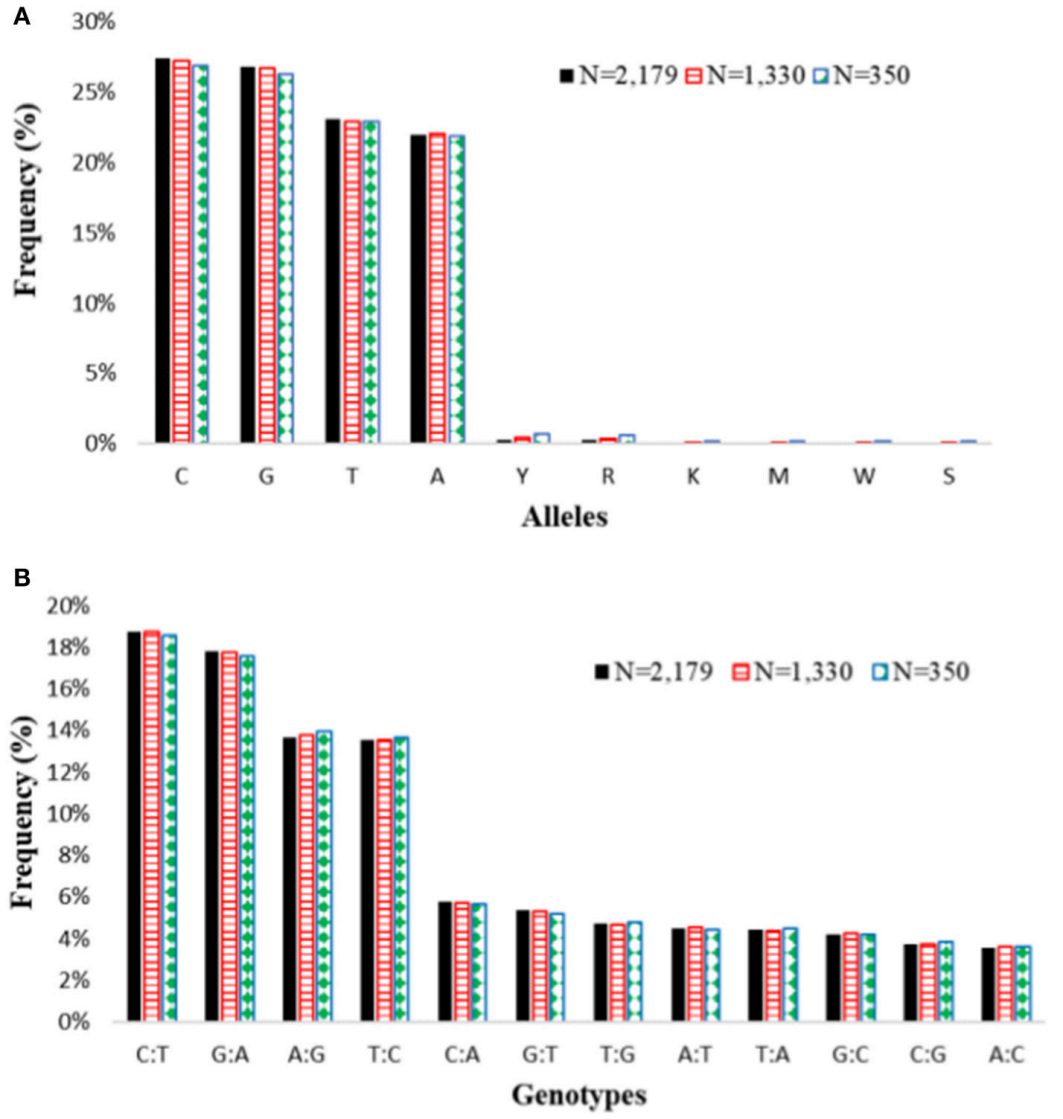
Summary of **(A)** allele frequencies and **(B)** genotype frequencies of the entire *O. glaberrima* accessions (*N* = 2,179) based on 3,834 polymorphic SNPs as compared with a core set of 1,330 accessions using 3,781 polymorphic SNPs, and a mini-core set of 350 accessions using 3,709 polymorphic SNPs. Nucleotide codes are as follows: A, adenine; C, cytosine; G, guanine; T, thymine; Y, C or T; R, A or G; K, G or T; M, A or C; W, A or T; S, G or C (http://www.bioinformatics.org/sms/iupac.html; accessed in Sept. 2017).

**Figure 7:**
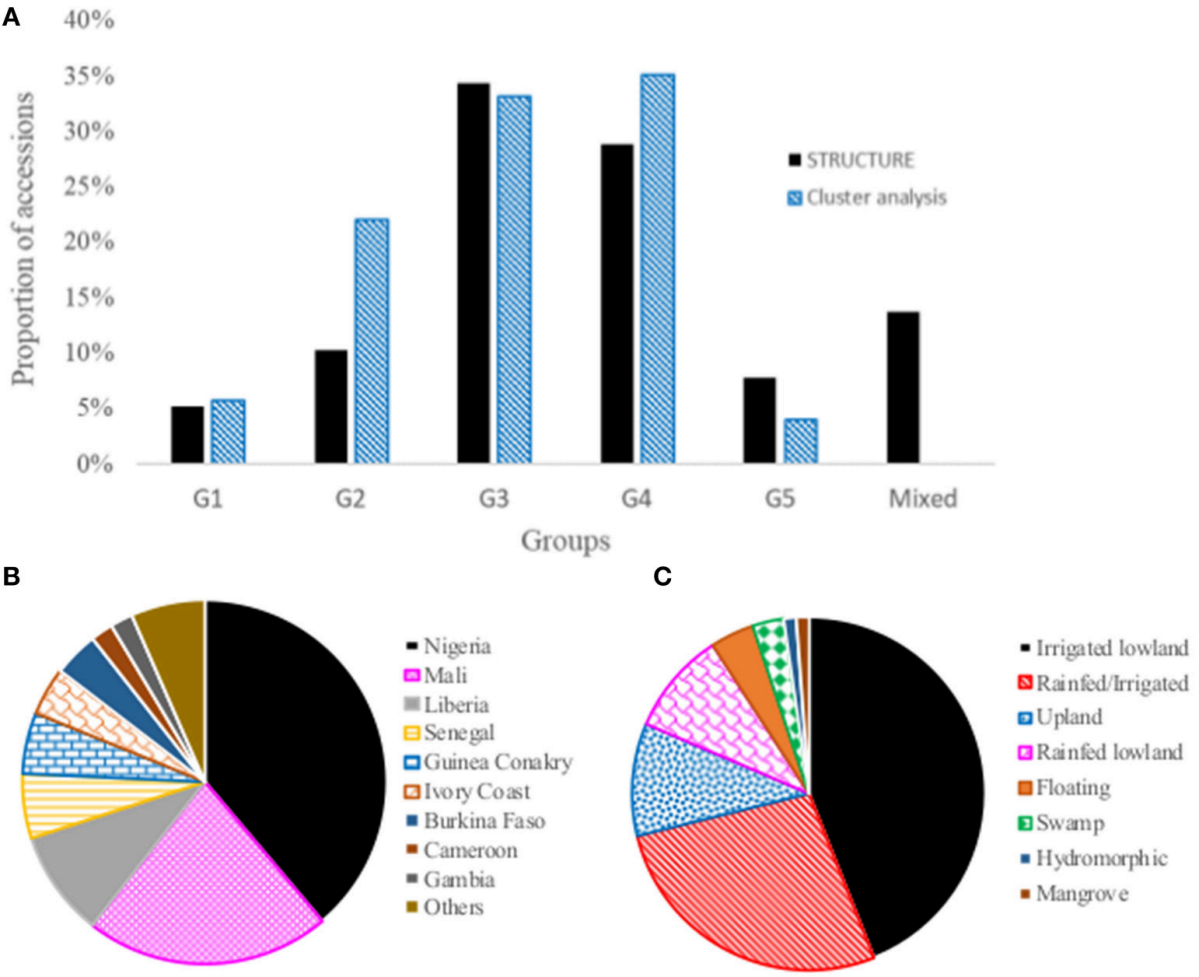
The distribution of accessions selected to represent mini-core collection by **(A)** groups predicted based on STRUCTURE and cluster analysis; **(B)** countries of origin, and **(C)** adaptation ecologies. See Supplementary Table S1 for details.

The genotypic data of the whole collection was compared to the data sets generated for the core and mini core sets for polymorphism, allele and genotype (combination of alleles) frequency, and genetic distance. For the 3,834 SNPs that were polymorphic in the whole *O. glaberrima* accessions, the reduction in sample size from 2,179 accessions to 1,330 accessions in the core and 350 accessions in the mini-core decreased the number of polymorphic SNPs only by 53 (1.4%) and 125 (3.3%), respectively. Both allele and genotype frequencies in the core and mini-core sets were the same as the whole set (Figure 6). As expected, however, genetic distance between pairs of accessions increased significantly in the core and mini-core sets (Figure 1, Supplemental Table S4). The proportion of pairs of accessions with genetic distance exceeding 0.10 was 90.5% for the whole set as compared with 95.3% in the core and 99.9% in the mini-core sets. The average phenotypic traits (number of tillers, days to heading, plant height, blade length, blade width, and panicle length) in the core and mini-core sets were basically the same as the whole *O. glaberrima* collection (Table 5). The proportion of accessions selected from each group predicted based on STRUCTURE and cluster analysis was not equal, but reflected the size of each group or cluster (Figure 7A). Using the group membership from STRUCTURE, the mini-core collection comprised of 18 accessions from group 1; 36 accessions from group 2; 120 accessions from group 3; 101 accessions from group 4; 27 accessions from group 5; and 48 accessions from the admixed group. Although the selected mini-core set of accessions represent 20 countries and all 9 ecologies, the majority of them originated from 9 countries (Figure 7B) and 6 ecologies (Figure 7C).

**Table 5:** Summary of seven phenotypic descriptors for the core and mini-core sets as compared to the whole *O. glaberrima* collection based on unreplicated data evaluation at the AfricaRice experimental station in Cotonou, Benin.

**Trait**	**Entries**	No. of entries with no missing phenotype^*^	**Minimum**	**Maximum**	**Mean**	**Standard deviation**	**Coefficient of variation**
Days to 50% heading (d)	All	2,047	63.0	183.0	112.7	21.6	19.1
	Core	1,221	63.0	183.0	117.4	22.1	18.8
	Mini-core	319	71.0	183.0	124.4	21.2	17.0
Total number of tillers	All	1,983	3.2	78.5	23.3	8.5	36.5
	Core	1,171	4.0	78.5	23.6	8.6	36.4
	Mini-core	301	6.8	77.0	24.5	9.1	37.3
Number of fertile tillers	All	1,975	1.0	44.0	16.0	6.8	42.7
	Core	1,167	1.0	43.4	15.3	6.8	44.4
	Mini-core	298	1.5	43.4	14.2	7.0	49.2
Plant height (cm)	All	1,980	82.8	182.0	130.4	15.8	12.2
	Core	1,167	82.8	182.0	131.3	16.5	12.6
	Mini-core	297	86.0	182.0	132.2	17.7	13.4
Panicle length (cm)	All	1,981	1.9	58.0	31.9	6.3	19.7
	Core	1,169	1.9	58.0	31.6	6.7	21.2
	Mini-core	299	13.4	58.0	30.5	6.7	21.9
Leaf blade length (cm)	All	1,990	16.7	75.6	37.0	8.3	22.4
	Core	1,175	16.9	70.1	36.5	8.6	23.5
	Mini-core	303	16.9	70.1	35.7	9.2	25.6
Leaf blade width (cm)	All	1,978	0.8	3.5	1.8	0.4	21.1
	Core	1,171	0.8	3.5	1.7	0.4	21.3
	Mini-core	302	0.9	2.6	1.6	0.3	20.1

Each accession was represented by mean values of 5 randomly selected plants.

* *The total number of accessions for the whole collection, core, and mini-core sets were 2,179, 1,330 and 350. Some accessions did not germinate; accessions with extremely small or large values were excluded from the phenotype data based on frequency distribution and boxplots*.

## Discussion

### Low genetic variation exists in the collection of *O. glaberrima*

Nearly 60% of the 27,560 SNPs used for genotyping 5 rice species was initially polymorphic within the *O. glaberrima* collection; however, 46% of the polymorphisms were due to just 44 accessions (Supplementary Table S1) that showed extremely different pattern as compared with all other *O. glaberrima* accessions (Supplementary Figure S2). It is possible that the 44 accessions may possess rare alleles that made them very different from the remaining 2,179 *O. glaberrima* accessions. However, those 44 accessions may also be natural introgressions involving *O. glaberrima* and another *Oryza* species, as has been reported in other studies (Jones et al., 1997; Semon et al., 2005; Orjuela et al., 2014). For example, Orjuela et al. (2014) reported some weedy type accessions that were intermediate between *O. glaberrima* and *O. barthii*, which were collected primarily in countries where the wild *O. barthii* grow near *O. glaberrima* fields. The presence of cultivated and wild species in the same area probably favored interspecific gene flow between two rice species, which resulted to the development of intermediate forms. Furthermore, we cannot rule out the possibility that other *Oryza* species were mislabeled as *O. glaberrima* during collection or at some stage during seed/DNA handling, DNA contamination, and/or genotyping error. These possible errors can be corrected with further study, including phenotypic evaluation of the 44 accessions in comparison with other widely used *O. glaberrima* accessions, and comparing the DArTseq data from the 44 accessions to data generated from other *Oryza* species. Due to such concerns, we presented detailed results on 2,179 accessions, excluding the 44 accessions listed in Supplementary Table S1.

When the 44 accessions described above were excluded, only 13.9% of the SNPs (3,834 of the 27,560 SNPs) were polymorphic within the remaining 2,179 *O. glaberrima* accessions (Table 2), which is lower than the polymorphism levels observed within *O. barthii* (18.9%) and *O. longistaminata* (30.3%) (MN Ndjiondjop, unpublished). In another study, we used the same set of 27,560 SNPs for genotyping 331 widely used rice genotypes in Africa, which includes 85 “New Rice for Africa (NERICA)” improved varieties, 11 “Advanced Rice for Africa (ARICA)” varieties, 62 *O. sativa* spp. japonica, and 172 *O. sativa* spp. indica genotypes. Sixty six percent of the SNPs (18,186 of the 27,560 SNPs) were polymorphic across all 331 genotypes, of which 15,020 SNP (82.6%) were physically mapped in to one of the rice chromosomes. Of the 15,020 physically mapped SNPs, nearly 37, 43, 48, and 49%, respectively, were polymorphic within ARICA, japonica, indica, and NERICA genotypes (MN Ndjiondjop, unpublished); therefore, the level of polymorphism observed in *O. sativa* and interspecific NERICA genotypes was from 2.5 to 3.5 times higher than that of *O. glaberrima*. Several previous molecular studies conducted on *O. glaberrima* have also reported a narrow genetic base compared to other *Oryza* species (Joshi et al., 2000; Ishii et al., 2001; Park et al., 2003; Semon et al., 2005; Kwon et al., 2006; Dramé et al., 2011; Li et al., 2011; Orjuela et al., 2014; Wang et al., 2014; Meyer et al., 2016). However, these previous studies were based on small numbers of accessions and/or markers, and the very large number of accessions and SNPs in the current study is much more definitive. The low levels of diversity confirmed in the present study may be due to a severe bottleneck that occurred during its domestication (Li et al., 2011; Wang et al., 2014; Meyer et al., 2016). Based on nucleotide diversity, the minor allele frequency spectrum, linkage disequilibrium (LD) and Tajima's *D*-values estimated from genome sequence data, Wang et al. (2014) suggested that the significant reduction in genetic diversity within *O. glaberrima* is a consequence of domestication, which is consistent with other studies reporting reduction in genetic diversity in domesticated crops as compared to their wild progenitors (Reif et al., 2005; Wright et al., 2005; Lam et al., 2010; Huang et al., 2012).

*O. glaberrima* is a selfing species with outcrossing rates ranging from 2 to 5% (Semon et al., 2005), even lower than many other selfing species (Barnaud et al., 2008). As a result, most accessions were expected to display less than the average observed heterozygosity expected in S_5_ breeding lines derived from biparental crosses (3.1%). This was seen with ~95% of the accessions, but the remaining ~4% (81 of 2,179 accessions) had observed heterozygosity exceeding 5% (Supplementary Table S1), which is higher than expected in the absence of human error. Some changes in allele frequencies may have occurred during seed regeneration, maintenance breeding, and possible contamination with seeds or pollen of other samples (Heckenberger et al., 2002; Warburton et al., 2010). These errors can easily be controlled with the development of quality control genotyping methods using a subset of 50 to 100 preselected markers following seed production (Semagn et al., 2012a; Ertiro et al., 2015), which will be addressed in another study.

### Genetic distance and population structure

Genetic distance is a measure of the genetic divergence between pairs of accessions or populations, with pairs that share many alleles having small genetic distance. Results from the present study showed highly variable levels of genetic distances between pairs of *O. glaberrima* accessions, with 1.5% of the pairs nearly identical, 8.0% of the pairs similar, 78.1% of the pairs moderately distant, and 12.4% of the pairs highly distant (Figure 1). Our results clearly suggest the presence of a low percentage of redundant accessions-that contribute very little to the observed genetic variation and genetic divergence in the *O. glaberrima* collection maintained by the AfricaRice genebank. Using cluster, principal component, and model-based population structure analyses, we observed five groups or clusters, which are generally consistent with their country of origin and somewhat less so with respect to their adaptation to the different rice growing ecologies (Figures 2–4, Supplementary Table S1); this classification agrees with previous studies (Semon et al., 2005; Orjuela et al., 2014).

Most accessions from Togo in the present study were collected from the Danyi Plateau and clustered with some Nigerian accessions in the second group. Despite the availability in Togo of improved rice varieties, farmers on the Danyi Plateau in the Togo Hills still cultivate solely *O. glaberrima* landraces because of good grain quality and palatability as well as better adaptation to soil with low fertility and high acidity (Mokuwa et al., 2014). These landraces produce heavier and larger seeds, which germinate better and produce more vigorous seedlings with deeper initial root system than smaller seeds (Roy et al., 1996). The high genetic divergence of the *O. glaberrima* accessions from Togo compared with all other countries, except Ghana, is evident from the *F*_ST_ values presented in Supplementary Table S3 and neighbor-joining tree constructed using the pairwise *F*_ST_ values (Supplementary Figure S6). Why some Nigerian accessions clustered with these divergent accessions from Togo, however, is unclear, but seed is easily traded and the two countries are not so distant. The clustering of accessions from these countries in to the same group may thus reflect historical trade routes.

Isolation by distance (Wright, 1943) refers to a decline in gene flow with an increase in geographical distance between pairs of populations. In such cases, pairs of accessions or populations sampled in close geographic proximity to each other are expected to be less genetically divergent (more genetically similar) than those that are geographically far away from each other. While most accessions in the current study were more similar within countries (Figures 2–4), there were several divergent pairs of accessions within the same countries, and thus less evidence for isolation by distance in the *O. glaberrima* germplasm used in the present study. This is in contrast to a previous study (Semon et al., 2005), and the larger numbers of accessions in the current study may also show evidence of migration via trade (which negates the decline in gene flow caused by distance). Most farmers in Africa trade seeds at nearby markets and with friends and relatives; this trade can cross country borders as can seed sold by regional seed traders. This may be particularly apparent in the fifth group that consisted of mixed accessions from multiple countries. Overall, pairwise *F*_ST_ values showed less differentiation between accessions from neighboring countries, with the exception of Gambia, which clustered closer to countries much further east (Supplementary Figure S6), possibly evidence of longer distance trade.

Knowledge of population structure is important for different purposes, including understanding the relative effect of evolutionary processes of migration/gene flow, mating system, selection, adaptation, and genetic drift on populations (including breeding populations) (Meirmans, 2012). Human activities significantly affect the dynamics of genetic differentiation in different ways, including changing the dynamics of gene flow, genetic drift and selection (Barnaud et al., 2008). AMOVA partitioned more of the molecular variation into groups (populations) based on country of origin (18.1%) than based on growing environment ecology (10.8%), indicating that migration (aided by humans) is a stronger (or faster acting) force on population structure than is selection for adaptation, drift, or mutation. The *O. glaberrima* germplasm used in the current study has not been selected for specific ecological adaptation, which may possibly be a reason for the lower observed genetic differentiation among ecologies than countries of origin. The proportion of molecular variation attributable among groups predicted from the cluster and model-based STRUCTURE was higher (27.3–28.9%) than among countries and ecologies. Therefore, there was more genetic divergence among the predicted groups that was not fully explained by geographical proximity or ecology. The development of core and mini-core collections and selection of parental lines for breeding programs need to consider such genetic divergence.

### Core and mini-core selection

The creation of core and mini-core germplasm collections that represent the maximum possible genetic diversity contained in the whole collection of a given species with a minimum of redundancy (Brown, 1989) is an efficient way to identify suitable variation for breeding and other purposes. A core collection can be created using various methods (Odong et al., 2013), including the random and stratified sampling methods that require subgroups created based on *a priori* knowledge (Brown, 1989); the maximization of the allelic diversity/richness method using MSTRAT (Gouesnard et al., 2001), Core Hunter (Thachuk et al., 2009; Beukelaer et al., 2012) and principal component scoring (Noirot et al., 1996); and the distance-based methods, such as Core Hunter, MLST (Perrier et al., 2003), and PowerCore (Kim et al., 2007). The choice of appropriate method depends on several criteria, including the purpose of the study (e.g., to capture most variation as much as possible in small accessions as possible vs. to optimize the chance of finding new alleles) (Odong et al., 2013), the computational speed of the method, and the need for *a priori* information (e.g., preselected markers, defined subgroups and/or sample size). In the maximization strategy, the main objective is to preserve the highest number of alleles, which is ideal for germplasm conservation, while the distance-based methods primarily aim to maintain most combinations of alleles in specific genotypes (maximize the combination of allelic diversity at the genome level), which is appropriate in breeding (Leroy et al., 2014). The distance-based methods have been strongly recommend for creating core collection, because they allow the simultaneous evaluation of all variables describing accessions, and also provide interpretable criteria/statistical support as compared with other methods (Odong et al., 2013). MLST has multiple advantages over other methods. First, it uses all markers simultaneously through a measure of dissimilarity instead of preselected subset of markers/alleles. Second, it considers the population structure in the data from the phylogenetic tree. Third, it requires neither *a priori* predefined subgroups nor *a priori* fixed sample size. Finally, it provides detailed statistical support indicators for users to choose an optimal core or mini-core sample size. MLST has been used in creating core collection in diverse species, including *Prunus avium* (Campoy et al., 2016), *Vigna unguiculata* (Egbadzor et al., 2014), *Coffea canephora* (Leroy et al., 2014), and *Olea europaea* (El Bakkali et al., 2013). Using MLST, we propose 1,330 and 350 *O. glaberrima* accessions to represent a core and mini-core collection, respectively (Supplementary Table S1). Core and mini-core sets can be developed using geographical origin, phenotypic traits, and biochemical and genetic markers; however, use of genetic distance computed from high density molecular markers from next generation sequencing technology is a promising method (Pessoa-Filho et al., 2010). The ability to measure sequence variation across the entire genome allows core sets to be chosen potentially based on all genes, and the effect of the environment does not influence the selection. If chosen randomly, between 5 and 20% of a core collection captures up to 70% of the genetic diversity observed in the whole collection (Brown, 1989; Van Hintum et al., 2000).

In rice, previously suggested core collections contain from 1% (Chung et al., 2009) to 15% (Liu et al., 2015) of the total number of accessions of the collections they represent. Using 435 alleles scored from 36 microsatellite markers, for example, only 1.4% (98 out of 6,912 accessions) were selected to represent a mini-core set of Indian rice collection conserved at the National Bureau of Plant Genetic Resources, New Delhi, India (Tiwari et al., 2015); the selected mini-core set captured 100% of the total number of alleles. Using 482 alleles from 15 microsatellite markers, only 4% (162 of 4,046 accessions) sampled from the National GeneBank of Rural Development Administration in the Republic of Korea captured 100% of the allelic variation (Zhao et al., 2010). Recently, Egea and colleagues used 14,392 SNPs from DArTseq for genotyping 417 garlic samples (*Allium sativum*), which allowed them in identifying 286 unique samples to represent a core collection (Egea et al., 2017). In barley, Munoz-Amatriain and colleagues recently used 7,842 SNPs to genotype 2,417 accessions sampled from the United States Department of Agriculture (USDA) National Small Grains Collection and created a mini-core set of 186 accessions that captured the majority of the allelic diversity present in the core collection (Munoz-Amatriain et al., 2014). The mini-core collection proposed in the present study is easily manageable for detailed phenotypic evaluation under field and controlled conditions for parent selection in developing new improved rice germplasm for target traits of interest, mining genes and genomic regions associated with target traits of economic importance using genome-wide association studies, and easy for wide germplasm distribution.

## Conclusions

The present study demonstrated the usefulness of DArTseq in effectively characterizing the genetic variation and population structure of *O. glaberrima* collection conserved at the AfricaRice genebank, and in creating a core and mini-core collections. Overall, *O. glaberrima* showed very low genetic variation, but clear population structure, primarily by countries of origin, and less so by ecology. The mini-core collection accounts ~16% of the whole *O. glaberrima* germplasm and represents all countries and ecologies of origin, and all groups predicted based on both cluster and population STRUCTURE analyses, proportionally to the size of each cluster. This mini-core collection will be highly valuable for various purposes, including allele mining and donor parent selection for developing new improved rice germplasm for target traits of interest.

## Data availability

All relevant results are included within this article and its additional files. The original DArTseq data are available from the corresponding author on request.

## Author contributions

MNN conceived, designed, and supervised the experiments, secured funding, and partly drafted the paper; ACG, SBK, DDT, and AGo were responsible for sample preparation, phenotyping, DNA extraction and/or compilation of passport information; KS analyzed the data and wrote most part of the paper; XP contributed to data analysis to create core and mini-core collection; MSo, MSi, and AGh provided valuable suggestions on the paper; MLW contributed to and edited the paper. All authors read and approved the paper.

### Conflict of interest statement

The authors declare that the research was conducted in the absence of any commercial or financial relationships that could be construed as a potential conflict of interest.
