# Supplementary material for: Genetic Variation and Population Structure of Oryza glaberrima and Development of a Mini-Core Collection Using DArTseq
Source: Front Plant Sci. 2017 Oct 17;8:1748. doi: 10.3389/fpls.2017.01748 (PMC5651524; doi:10.3389/fpls.2017.01748)
Supplement: Supplementary file 5 [file SupplementaryFigures.PDF]

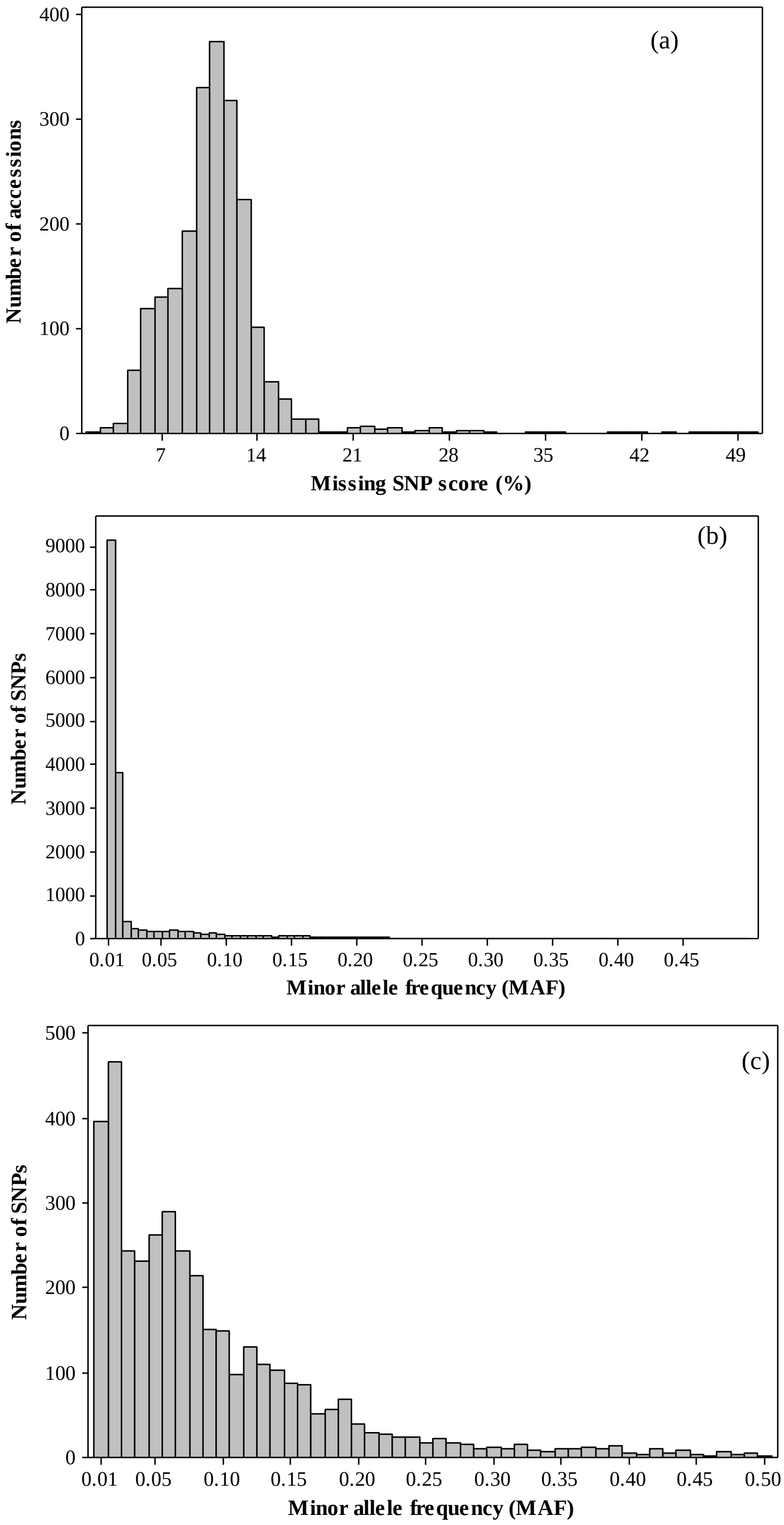

Supplementary Figure S1 Frequency distribution of (a) missing SNP calls of the *O. glaberrima* accessions selected for imputation; (b) minor allele frequency (MAF) of the 16,532 imputed SNPs that were polymorphic among the 2,223 accessions; and (c) MAF of the 3,834 imputed SNPs that were polymorphic among the 2,179 accessions (after excluding 44 accessions that appeared to be very different from all other accessions).

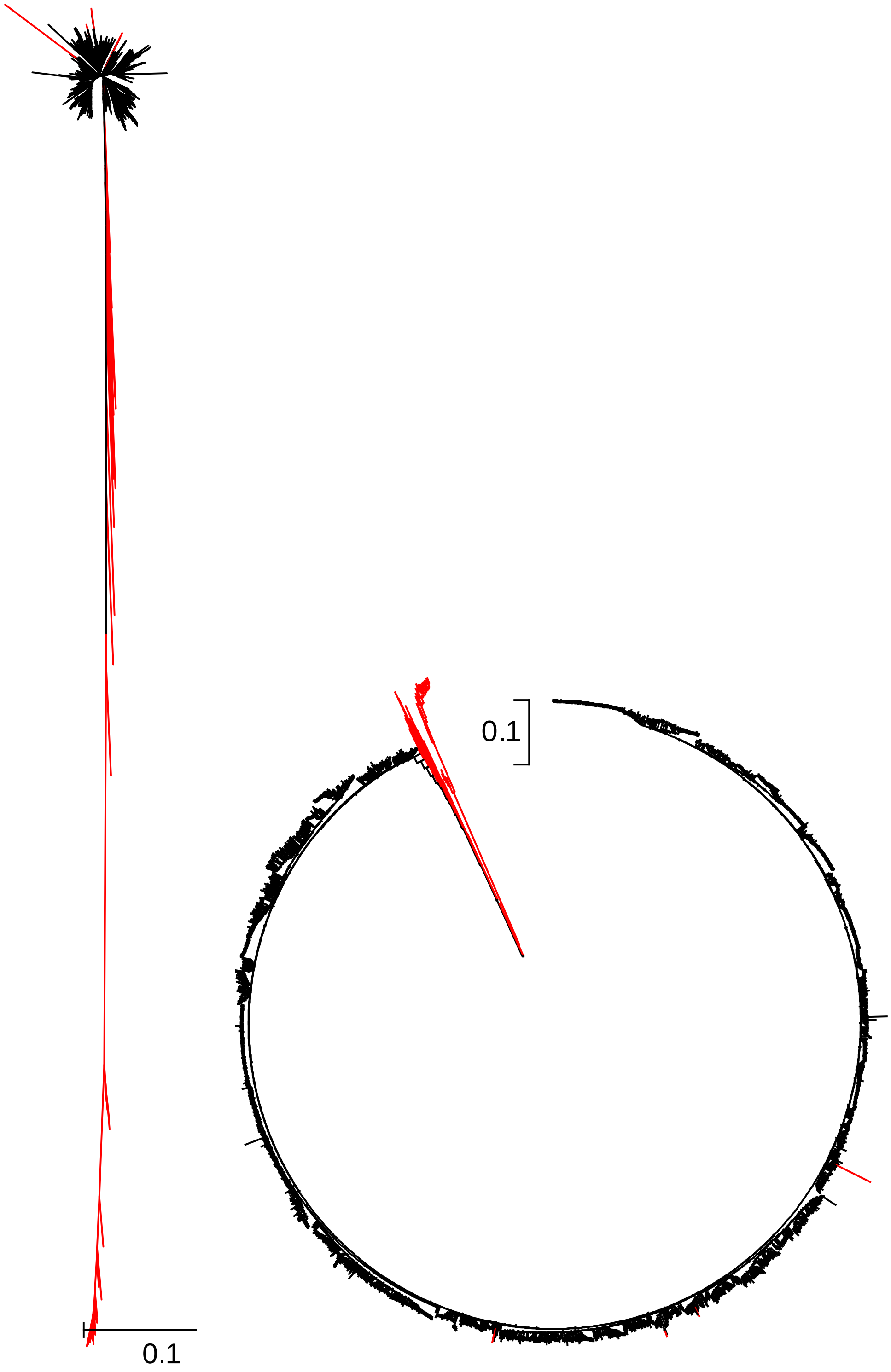

Supplementary Figure S2 Neighbor-joining tree of 2,223 *O. glaberrima* accessions based on 16,532 SNPs: radial tree (left) and circular tree (right). The 44 radically different accessions that affected the analyses are in red color. See Supplementary Table S1 for list of accessions and their origin.

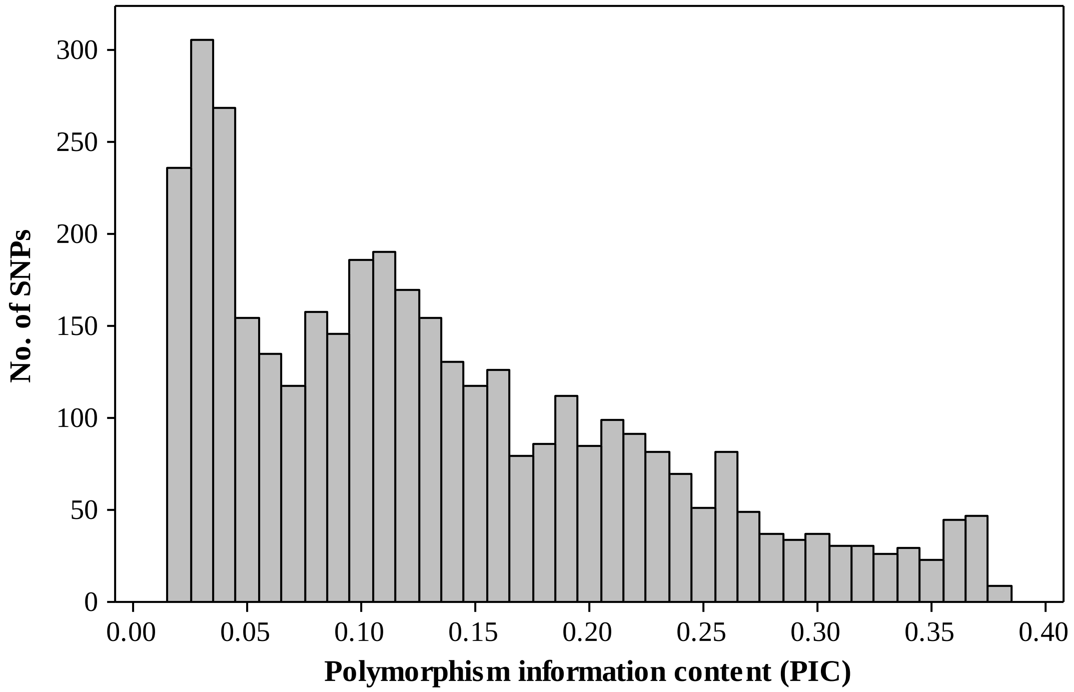

Supplementary Figure S3 Frequency distribution of polymorphism information content (PIC) of 3,834 SNPs used for genotyping 2,179 *O. glaberrima* accessions.

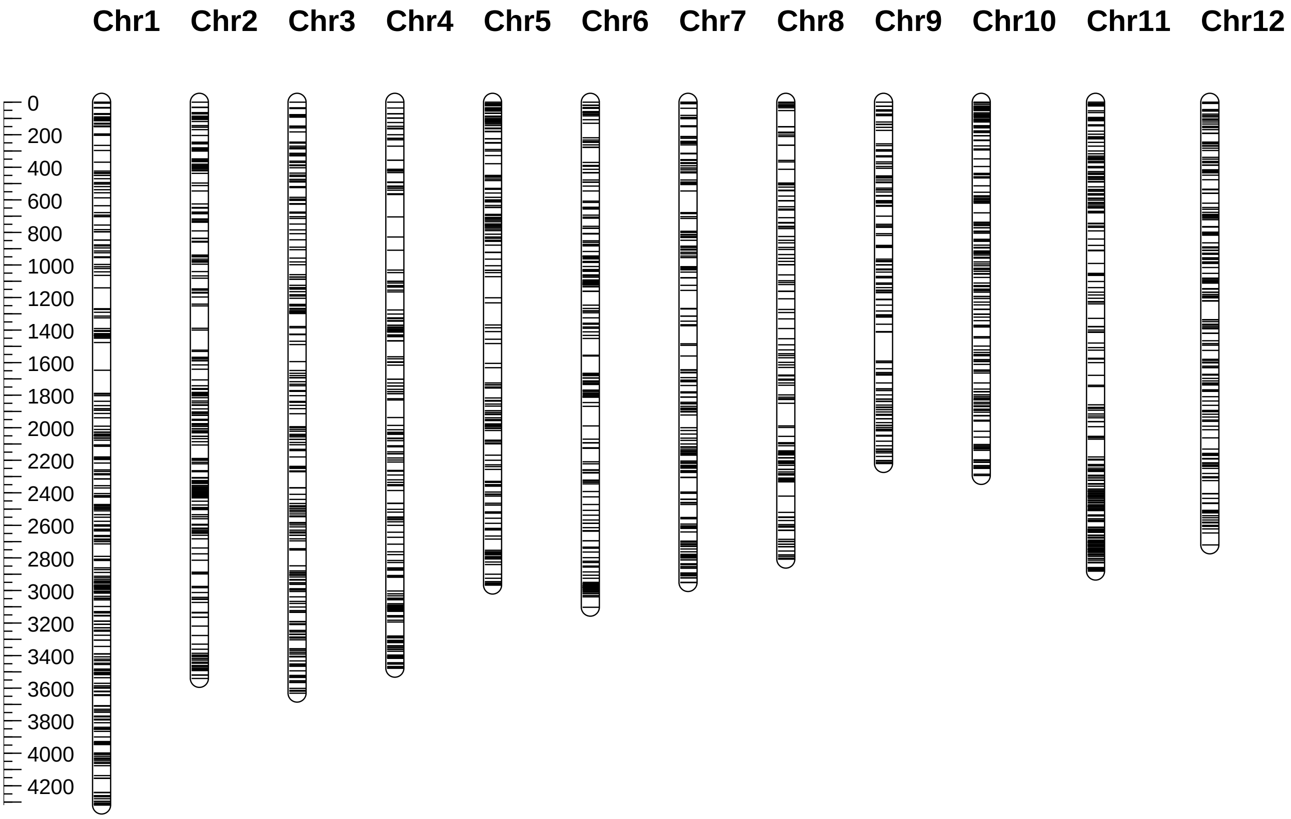

Supplementary Figure S4. The chromosomal distribution and position of the 3,834 polymorphic SNPs used in characterizing 2,179 *O. glaberrima* accessions. Map position (x 10,000 bp) is shown on the left side. For each chromosome, the horizontal line represents a single SNP; the black shaded regions indicate high marker density due to presence of multiple SNPs with small physical distance, while the white regions indicate lower marker density in that interval.

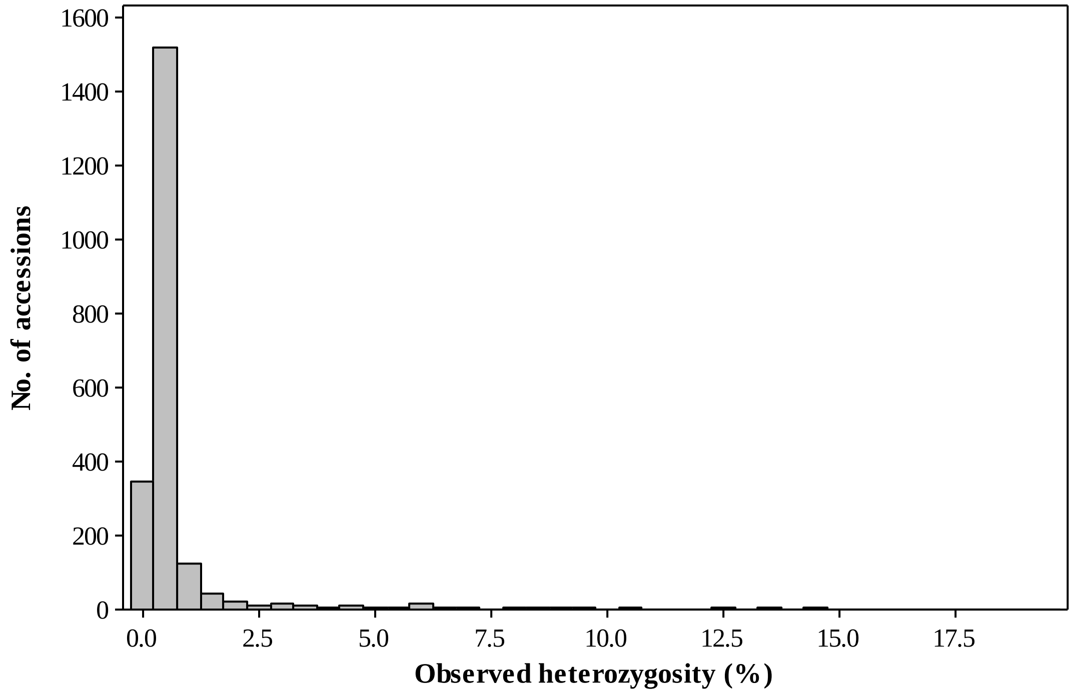

Supplementary Figure S5 Frequency distribution of residual heterozygosity for 2,179 *O. glaberrima* accessions based on 3,834 polymorphic SNPs. See observed heterozygosity of each accession in Supplementary Table S1.

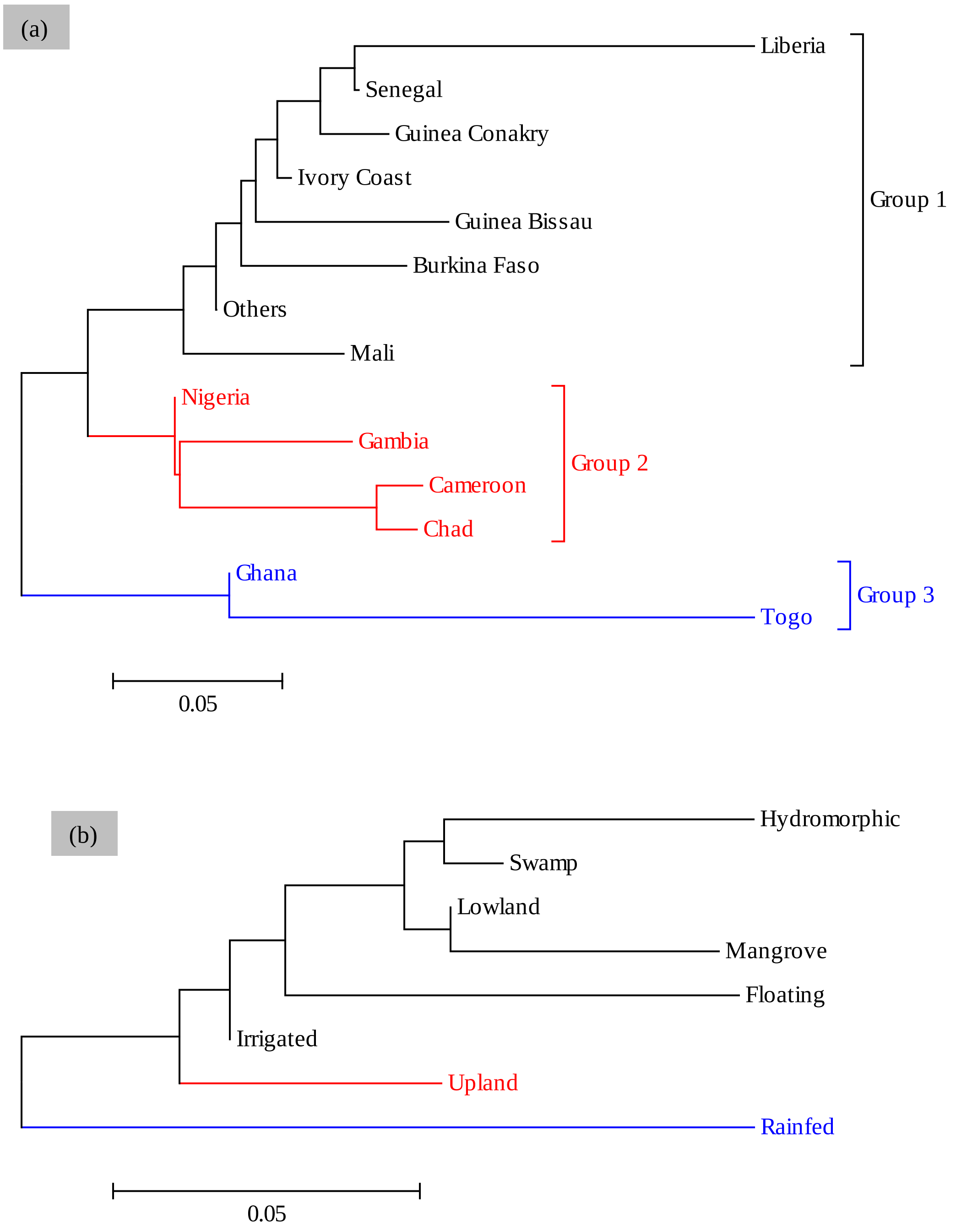

Supplementary Figure S6 Neighbor joining tree based on F_{ST} values estimated between pairs of (a) countries and (b) ecologies based on 2,179 *O. glaberrima* accessions genotyped with 3,834 SNPs. Only countries with >14 accessions were shown here; those with <14 accessions were combined under "others".

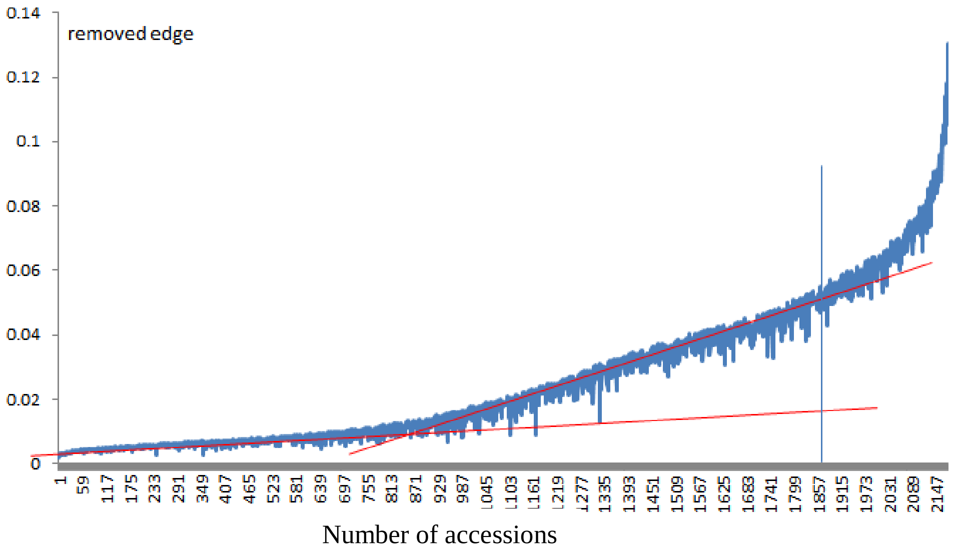

Supplementary Figure S7 Plot of the number of accessions vs. removed edge obtained using the maximum length subtree method implemented in DARwin v6.0.14. The first part of the curve shown in red line is approximately linear with a relatively low slope until it reaches 849 accessions (the intersection of the two red lines), which suggests that a core set of 1,330 accessions (bottom figure) can be created by excluding the first set of 849 accessions that are likely to be redundant.

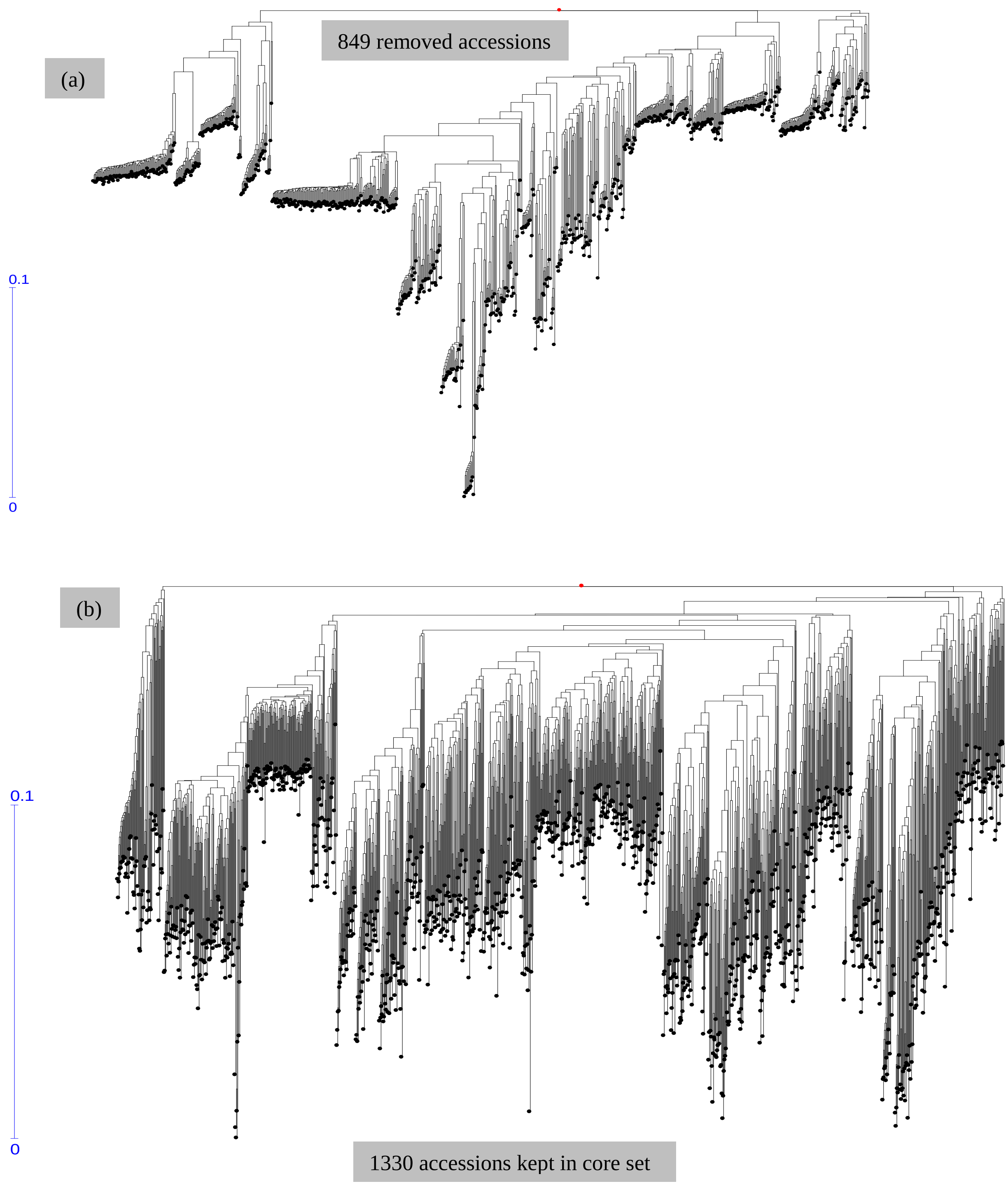

Supplementary Figure S8 Neighbor joining tree for (a) 849 redundant accessions and (b) 1,330 accessions selected for a core set based on 3,834 polymorphic SNPs. See Supplementary Table S1 for list of removed and retained accessions.
